# Persistent interaction patterns across social media platforms and over time

**DOI:** 10.1038/s41586-024-07229-y

**Published:** 2024-03-20

**Authors:** Michele Avalle, Niccolò Di Marco, Gabriele Etta, Emanuele Sangiorgio, Shayan Alipour, Anita Bonetti, Lorenzo Alvisi, Antonio Scala, Andrea Baronchelli, Matteo Cinelli, Walter Quattrociocchi

**Affiliations:** 1 Department of Computer Science, Sapienza University of Rome, Rome, Italy; 2 Department of Social Sciences and Economics, Sapienza University of Rome, Rome, Italy; 3 Department of Communication and Social Research, Sapienza University of Rome, Rome, Italy; 4 Institute of Complex Systems, CNR, Rome, Italy; 5 Department of Mathematics, City University of London, London, UK; 6 The Alan Turing Institute, London, UK

**Keywords:** Mathematics and computing, Social sciences

## Abstract

Growing concern surrounds the impact of social media platforms on public discourse^1–4^ and their influence on social dynamics^5–9^, especially in the context of toxicity^10–12^. Here, to better understand these phenomena, we use a comparative approach to isolate human behavioural patterns across multiple social media platforms. In particular, we analyse conversations in different online communities, focusing on identifying consistent patterns of toxic content. Drawing from an extensive dataset that spans eight platforms over 34 years—from Usenet to contemporary social media—our findings show consistent conversation patterns and user behaviour, irrespective of the platform, topic or time. Notably, although long conversations consistently exhibit higher toxicity, toxic language does not invariably discourage people from participating in a conversation, and toxicity does not necessarily escalate as discussions evolve. Our analysis suggests that debates and contrasting sentiments among users significantly contribute to more intense and hostile discussions. Moreover, the persistence of these patterns across three decades, despite changes in platforms and societal norms, underscores the pivotal role of human behaviour in shaping online discourse.

## Abstract (web summary)

Long conversations online consistently exhibit higher toxicity, yet toxic language does not invariably discourage people from participating in a conversation, and toxicity does not necessarily escalate as discussions evolve.

## Main

The advent and proliferation of social media platforms have not only transformed the landscape of online participation^2^ but have also become integral to our daily lives, serving as primary sources for information, entertainment and personal communication^13,14^. Although these platforms offer unprecedented connectivity and information exchange opportunities, they also present challenges by entangling their business models with complex social dynamics, raising substantial concerns about their broader impact on society. Previous research has extensively addressed issues such as polarization, misinformation and antisocial behaviours in online spaces^5,7,12,15–17^, revealing the multifaceted nature of social media’s influence on public discourse. However, a considerable challenge in understanding how these platforms might influence inherent human behaviours lies in the general lack of accessible data^18^. Even when researchers obtain data through special agreements with companies like Meta, it may not be enough to clearly distinguish between inherent human behaviours and the effects of the platform’s design^3,4,8,9^. This difficulty arises because the data, deeply embedded in platform interactions, complicate separating intrinsic human behaviour from the influences exerted by the platform’s design and algorithms.

Here we address this challenge by focusing on toxicity, one of the most prominent aspects of concern in online conversations. We use a comparative analysis to uncover consistent patterns across diverse social media platforms and timeframes, aiming to shed light on toxicity dynamics across various digital environments. In particular, our goal is to gain insights into inherently invariant human patterns of online conversations.

The lack of non-verbal cues and physical presence on the web can contribute to increased incivility in online discussions compared with face-to-face interactions^19^. This trend is especially pronounced in online arenas such as newspaper comment sections and political discussions, where exchanges may degenerate into offensive comments or mockery, undermining the potential for productive and democratic debate^20,21^. When exposed to such uncivil language, users are more likely to interpret these messages as hostile, influencing their judgement and leading them to form opinions based on their beliefs rather than the information presented and may foster polarized perspectives, especially among groups with differing values^22^. Indeed, there is a natural tendency for online users to seek out and align with information that echoes their pre-existing beliefs, often ignoring contrasting views^6,23^. This behaviour may result in the creation of echo chambers, in which like-minded individuals congregate and mutually reinforce shared narratives^5,24,25^. These echo chambers, along with increased polarization, vary in their prevalence and intensity across different social media platforms^1^, suggesting that the design and algorithms of these platforms, intended to maximize user engagement, can substantially shape online social dynamics. This focus on engagement can inadvertently highlight certain behaviours, making it challenging to differentiate between organic user interaction and the influence of the platform’s design. A substantial portion of current research is devoted to examining harmful language on social media and its wider effects, online and offline^10,26^. This examination is crucial, as it reveals how social media may reflect and amplify societal issues, including the deterioration of public discourse. The growing interest in analysing online toxicity through massive data analysis coincides with advancements in machine learning capable of detecting toxic language^27^. Although numerous studies have focused on online toxicity, most concentrate on specific platforms and topics^28,29^. Broader, multiplatform studies are still limited in scale and reach^12,30^. Research fragmentation complicates understanding whether perceptions about online toxicity are accurate or misconceptions^31^. Key questions include whether online discussions are inherently toxic and how toxic and non-toxic conversations differ. Clarifying these dynamics and how they have evolved over time is crucial for developing effective strategies and policies to mitigate online toxicity.

Our study involves a comparative analysis of online conversations, focusing on three dimensions: time, platform and topic. We examine conversations from eight different platforms, totalling about 500 million comments. For our analysis, we adopt the toxicity definition provided by the Perspective API, a state-of-the-art classifier for the automatic detection of toxic speech. This API considers toxicity as “a rude, disrespectful or unreasonable comment likely to make someone leave a discussion”. We further validate this definition by confirming its consistency with outcomes from other detection tools, ensuring the reliability and comparability of our results. The concept of toxicity in online discourse varies widely in the literature, reflecting its complexity, as seen in various studies^32–34^. The efficacy and constraints of current machine-learning-based automated toxicity detection systems have recently been debated^11,35^. Despite these discussions, automated systems are still the most practical means for large-scale analyses.

Here we analyse online conversations, challenging common assumptions about their dynamics. Our findings reveal consistent patterns across various platforms and different times, such as the heavy-tailed nature of engagement dynamics, a decrease in user participation and an increase in toxic speech in lengthier conversations. Our analysis indicates that, although toxicity and user participation in debates are independent variables, the diversity of opinions and sentiments among users may have a substantial role in escalating conversation toxicity.

To obtain a comprehensive picture of online social media conversations, we analysed a dataset of about 500 million comments from Facebook, Gab, Reddit, Telegram, Twitter, Usenet, Voat and YouTube, covering diverse topics and spanning over three decades (a dataset breakdown is shown in Table 1 and Supplementary Table 1; for details regarding the data collection, see the ‘Data collection’ section of the Methods).

**Table 1:** Dataset breakdown

Dataset	Time range	Comments	Threads	Users	Toxicity
Facebook brexit	31 Dec 2015 to 29 Jul 2016	464,764	4,241	252,156	0.06
Facebook news	9 Sep 2009 to 18 Aug 2016	362,718,451	6,898,312	60,235,461	0.06
Facebook vaccines	2 Jan 2010 to 17 Jul 2017	2,064,980	153,137	387,084	0.04
Gab feed	10 Aug 2016 to 29 Oct 2018	14,641,433	3,764,443	166,833	0.13
Reddit climate change	1 Jan 2018 to 12 Dec 2022	70,648	5,057	26,521	0.07
Reddit conspiracy	1 Jan 2018 to 8 Dec 2022	777,393	35,092	92,678	0.07
Reddit news	1 Jan 2018 to 31 Dec 2018	389,582	7,798	109,860	0.09
Reddit science	1 Jan 2018 to 11 Dec 2022	549,543	28,330	211,546	0.01
Reddit vaccines	1 Jan 2018 to 6 Nov 2022	66,457	4,539	5,192	0.04
Telegram conspiracy	30 Aug 2019 to 20 Dec 2022	1,416,482	32,592	150,251	0.12
Telegram news	9 Apr 2018 to 20 Dec 2022	724,482	28,288	16,716	0.02
Telegram politics	4 Aug 2017 to 19 Dec 2022	491,294	27,749	6,132	0.04
Twitter climate change	1 Jan 2020 to 10 Jan 2023	9,709,855	130,136	3,577,890	0.07
Twitter news	1 Jan 2020 to 29 Nov 2022	9,487,587	97,797	1,710,213	0.07
Twitter vaccines	23 Jan 2010 to 25 Jan 2023	49,437,212	125,667	11,857,050	0.08
Usenet conspiracy	1 Sep 1994 to 30 Dec 2005	284,838	72,655	48,224	0.05
Usenet news	5 Dec 1992 to 31 Dec 2005	621,084	169,036	76,620	0.09
Usenet politics	29 Jun 1992 to 31 Dec 2005	2,657,772	625,945	209,905	0.08
Usenet talk	13 Feb 1989 to 31 Dec 2005	2,103,939	328,009	156,542	0.06
Voat conspiracy	9 Jan 2018 to 25 Dec 2020	1,024,812	99,953	27,641	0.10
Voat news	21 Nov 2013 to 25 Dec 2020	1,397,955	170,801	88,434	0.19
Voat politics	19 Jun 2014 to 25 Dec 2020	1,083,932	143,103	66,424	0.19
YouTube climate change	16 Mar 2014 to 28 Feb 2022	846,300	9,022	436,246	0.06
YouTube news	13 Feb 2006 to 8 Feb 2022	20,536,162	107,880	4,310,827	0.07
YouTube vaccines	31 Jan 2020 to 24 Oct 2021	2,648,909	14,147	902,340	0.04

Toxicity represents the fraction of toxic comments in the dataset, where a comment is considered toxic if its toxicity score is greater than 0.6.

Our analysis aims to comprehensively compare the dynamics of diverse social media accounting for human behaviours and how they evolved. In particular, we first characterize conversations at a macroscopic level by means of their engagement and participation, and we then analyse the toxicity of conversations both after and during their unfolding. We conclude the paper by examining potential drivers for the emergence of toxic speech.

## Conversations on different platforms

**Fig. 1:**
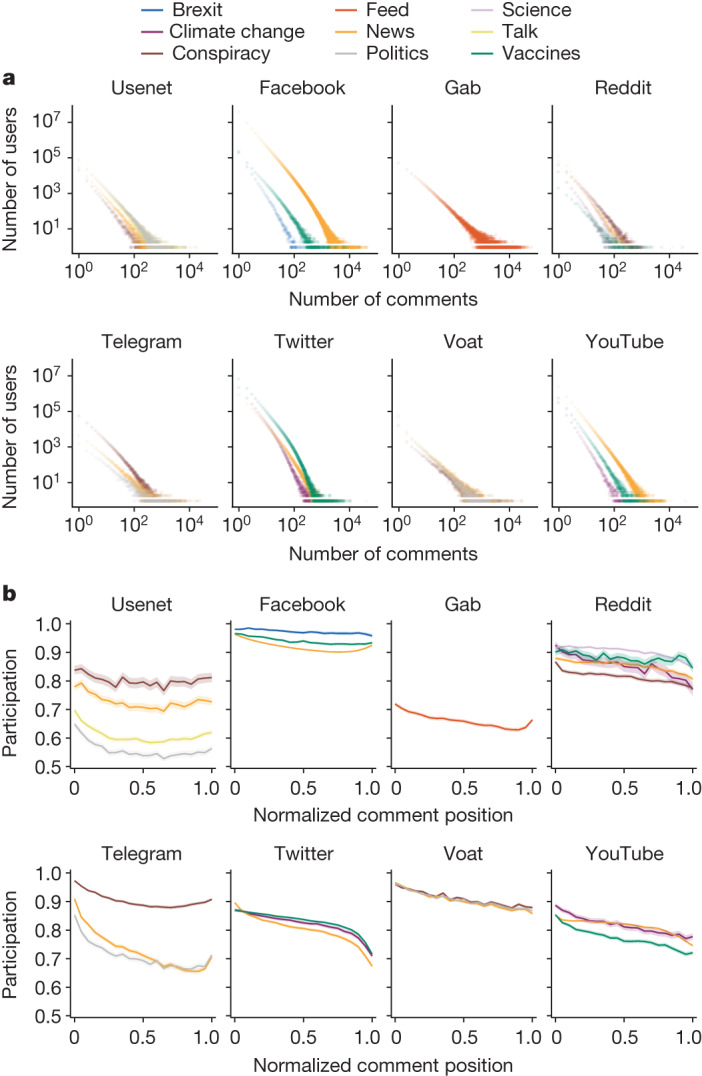
General characteristics of online conversations. **a**, The distributions of user activity in terms of comments posted for each platform and each topic. **b**, The mean user participation as conversations evolve. For each dataset, participation is computed for the threads belonging to the size interval [0.7–1] (Supplementary Table 2). Trends are reported with their 95% confidence intervals. The *x* axis represents the normalized position of comment intervals in the threads.

**Extended Data Fig. 1:**
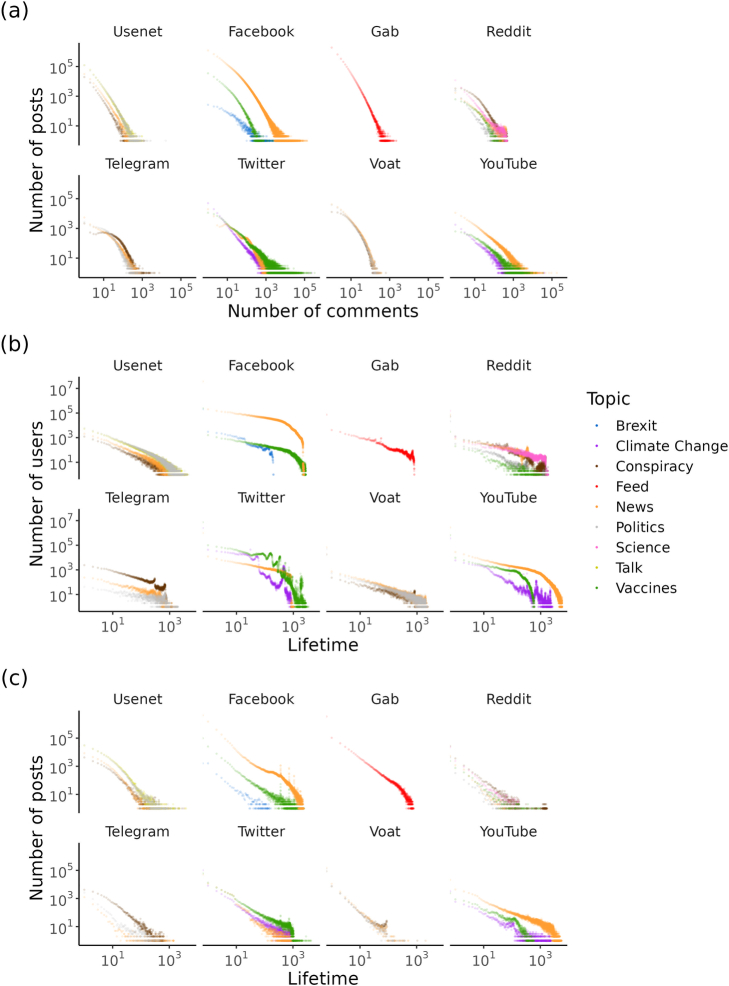
General characteristics of online conversations. **a**. Distributions of conversation length (number of comments in a thread). **b**. Distributions of the time duration (days) of user activity on a platform for each platform and each topic. **c**. Time duration (days) distributions of threads. Colour-coded legend on the side.

This section provides an overview of online conversations by considering user activity and thread size metrics. We define a conversation (or a thread) as a sequence of comments that follow chronologically from an initial post. In Fig. 1a and Extended Data Fig. 1, we observe that, across all platforms, both user activity (defined as the number of comments posted by the user) and thread length (defined as the number of comments in a thread) exhibit heavy-tailed distributions. The summary statistics about these distributions are reported in Supplementary Tables 1 and 2.

Consistent with previous studies^36,37^ our analysis shows that the macroscopic patterns of online conversations, such as the distribution of users/threads activity and lifetime, are consistent across all datasets and topics (Supplementary Tables 1–4). This observation holds regardless of the specific features of the diverse platforms, such as recommendation algorithms and moderation policies (described in the ‘Content moderation policies’ of the Methods), as well as other factors, including the user base and the conversation topics. We extend our analysis by examining another aspect of user activity within conversations across all platforms. To do this, we introduce a metric for the participation of users as a thread evolves. In this analysis, threads are filtered to ensure sufficient length as explained in the ‘Logarithmic binning and conversation size’ section of the Methods.

The participation metric, defined over different conversation intervals (that is, 0–5% of the thread arranged in chronological order, 5–10%, and so on), is the ratio of the number of unique users to the number of comments in the interval. Considering a fixed number of comments *c*, smaller values of participation indicate that fewer unique users are producing *c* comments in a segment of the conversation. In turn, a value of participation equal to 1 means that each user is producing one of the *c* comments, therefore obtaining the maximal homogeneity of user participation. Our findings show that, across all datasets, the participation of users in the evolution of conversations, averaged over almost all considered threads, is decreasing, as indicated by the results of Mann–Kendall test—a nonparametric test assessing the presence of a monotonic upward or downward tendency—shown in Extended Data Table 1. This indicates that fewer users tend to take part in a conversation as it evolves, but those who do are more active (Fig. 1b). Regarding patterns and values, the trends in user participation for various topics are consistent across each platform. According to the Mann–Kendall test, the only exceptions were Usenet Conspiracy and Talk, for which an ambiguous trend was detected. However, we note that their regression slopes are negative, suggesting a decreasing trend, even if with a weaker effect. Overall, our first set of findings highlights the shared nature of certain online interactions, revealing a decrease in user participation over time but an increase in activity among participants. This insight, consistent across most platforms, underscores the dynamic interplay between conversation length, user engagement and topic-driven participation.

## Conversation size and toxicity

**Fig. 2:**
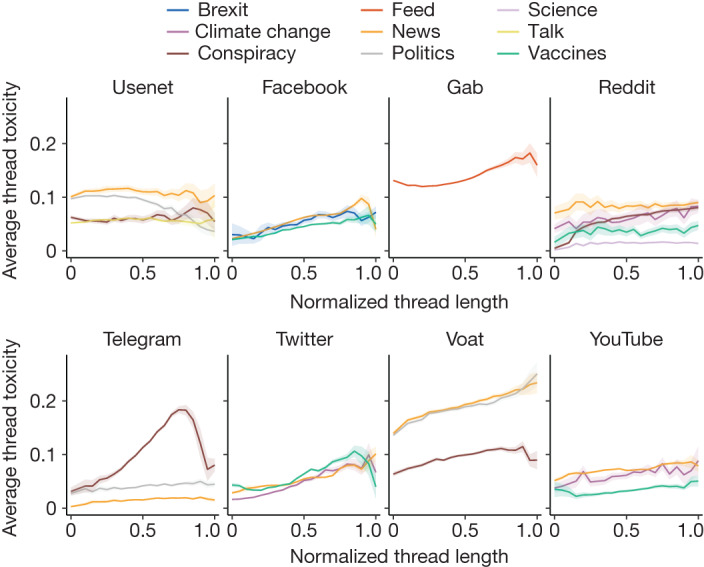
Toxicity increases with conversation size. The mean fraction of toxic comments in conversations versus conversation size for each dataset. Trends represent the mean toxicity over each size interval and their 95% confidence interval. Size ranges are normalized to enable visual comparison of the different trends.

**Extended Data Fig. 2:**
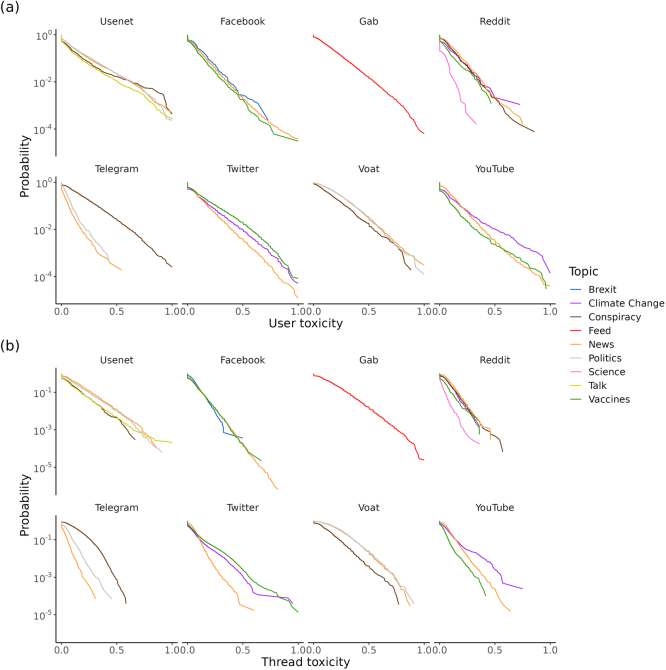
Extremely toxic authors and conversations are rare. **a**. Complementary cumulative distribution functions (CCDFs) of the toxicity of authors who posted more than 10 comments. Toxicity is defined as usual as the fraction of toxic comments over the total of comments posted by a user. **b**. CCDFs of the toxicity of conversations containing more than 10 comments. Colour-coded legend on the side.

To detect the presence of toxic language, we used Google’s Perspective API^34^, a state-of-the-art toxicity classifier that has been used extensively in recent literature^29,38^. Perspective API defines a toxic comment as “A rude, disrespectful, or unreasonable comment that is likely to make people leave a discussion”. On the basis of this definition, the classifier assigns a toxicity score in the [0,1] range to a piece of text that can be interpreted as an estimate of the likelihood that a reader would perceive the comment as toxic (https://developers.perspectiveapi.com/s/about-the-api-score). To define an appropriate classification threshold, we draw from the existing literature^39^, which uses 0.6 as the threshold for considering a comment as toxic. A robustness check of our results using different threshold and classification tools is reported in the ‘Toxicity detection and validation of employed models’ section of the Methods, together with a discussion regarding potential shortcomings deriving from automatic classifiers. To further investigate the interplay between toxicity and conversation features across various platforms, our study first examines the prevalence of toxic speech in each dataset. We then analyse the occurrence of highly toxic users and conversations. Lastly, we investigate how the length of conversations correlates with the probability of encountering toxic comments. First of all, we define the toxicity of a user as the fraction of toxic comments that she/he left. Similarly, the toxicity of a thread is the fraction of toxic comments it contains. We begin by observing that, although some toxic datasets exist on unmoderated platforms such as Gab, Usenet and Voat, the prevalence of toxic speech is generally low. Indeed, the percentage of toxic comments in each dataset is mostly below 10% (Table 1). Moreover, the complementary cumulative distribution functions illustrated in Extended Data Fig. 2 show that the fraction of extremely toxic users is very low for each dataset (in the range between 10^−3^ and 10^−4^), and the majority of active users wrote at least one toxic comment, as reported in Supplementary Table 5, therefore suggesting that the overall volume of toxicity is not a phenomenon limited to the activity of very few users and localized in few conversations. Indeed, the number of users versus their toxicity decreases sharply following an exponential trend. The toxicity of threads follows a similar pattern. To understand the association between the size and toxicity of a conversation, we start by grouping conversations according to their length to analyse their structural differences^40^. The grouping is implemented by means of logarithmic binning (see the ‘Logarithmic binning and conversation size’ section of the Methods) and the evolution of the average fraction of toxic comments in threads versus the thread size intervals is reported in Fig. 2. Notably, the resulting trends are almost all increasing, showing that, independently of the platform and topic, the longer the conversation, the more toxic it tends to be.

We assessed the increase in the trends by both performing linear regression and applying the Mann–Kendall test to ensure the statistical significance of our results (Extended Data Table 2). To further validate these outcomes, we shuffled the toxicity labels of comments, finding that trends are almost always non-increasing when data are randomized. Furthermore, the *z*-scores of the regression slopes indicate that the observed trends deviate from the mean of the distributions resulting from randomizations, being at least 2 s.d. greater in almost all cases. This provides additional evidence of a remarkable difference from randomness. The only decreasing trend is Usenet Politics. Moreover, we verified that our results are not influenced by the specific number of bins as, after estimating the same trends again with different intervals, we found that the qualitative nature of the results remains unchanged. These findings are summarized in Extended Data Table 2. These analyses have been validated on the same data using a different threshold for identifying toxic comments and on a new dataset labelled with three different classifiers, obtaining similar results (Extended Data Fig. 5, Extended Data Table 5, Supplementary Fig. 1 and Supplementary Table 8). Finally, using a similar approach, we studied the toxicity content of conversations versus their lifetime—that is, the time elapsed between the first and last comment. In this case, most trends are flat, and there is no indication that toxicity is generally associated either with the duration of a conversation or the lifetime of user interactions (Extended Data Fig. 4).

## Conversation evolution and toxicity

**Fig. 3:**
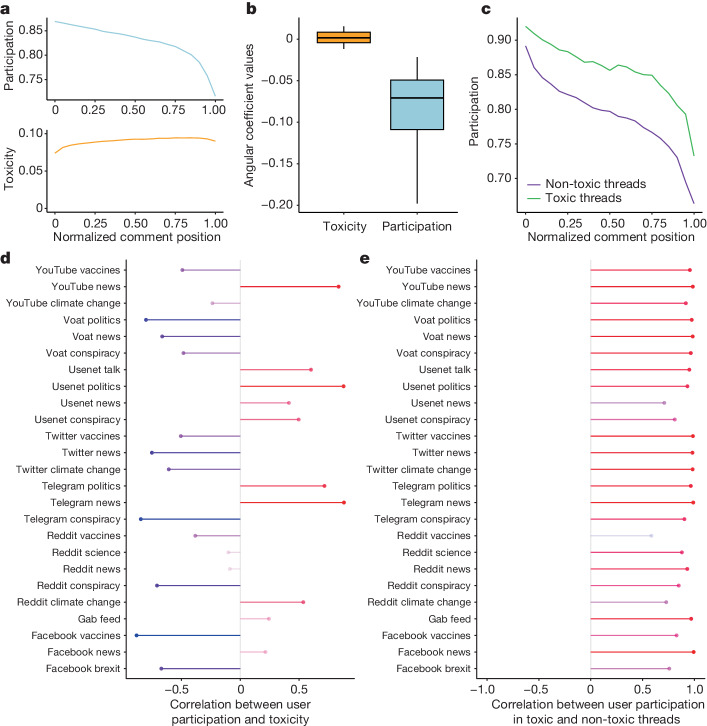
Participation of users is not dependent on toxicity. **a**, Examples of a typical trend in averaged user participation (top) and toxicity (bottom) versus the normalized position of comment intervals in the threads (Twitter news dataset). **b**, Box plot distributions of toxicity (*n* = 25, minimum = −0.012, maximum = 0.015, lower whisker = −0.012, quartile 1 (Q1) = − 0.004, Q2 = 0.002, Q3 = 0.008, upper whisker = 0.015) and participation (*n* = 25, minimum = −0.198, maximum = −0.022, lower whisker = −0.198, Q1 = − 0.109, Q2 = − 0.071, Q3 = − 0.049, upper whisker = −0.022) trend slopes for all datasets, as resulting from linear regression. **c**, An example of user participation in toxic and non-toxic thread sets (Twitter news dataset). **d**, Pearson’s correlation coefficients between user participation and toxicity trends for each dataset. **e**, Pearson’s correlation coefficients between user participation in toxic and non-toxic threads for each dataset.

**Extended Data Fig. 3:**
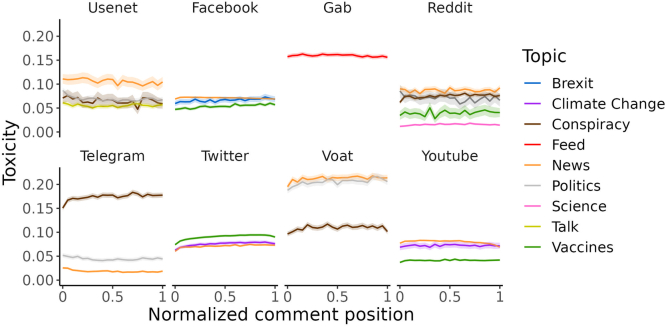
User toxicity as conversations evolve. Mean fraction of toxic comments as conversations progress. The x-axis represents the normalized position of comment intervals in the threads. For each dataset, toxicity is computed in the thread size interval [0.7−1] (see main text and Tab. S2 in SI). Trends are reported with their 95% confidence interval. Colour-coded legend on the side.

**Extended Data Fig. 4:**
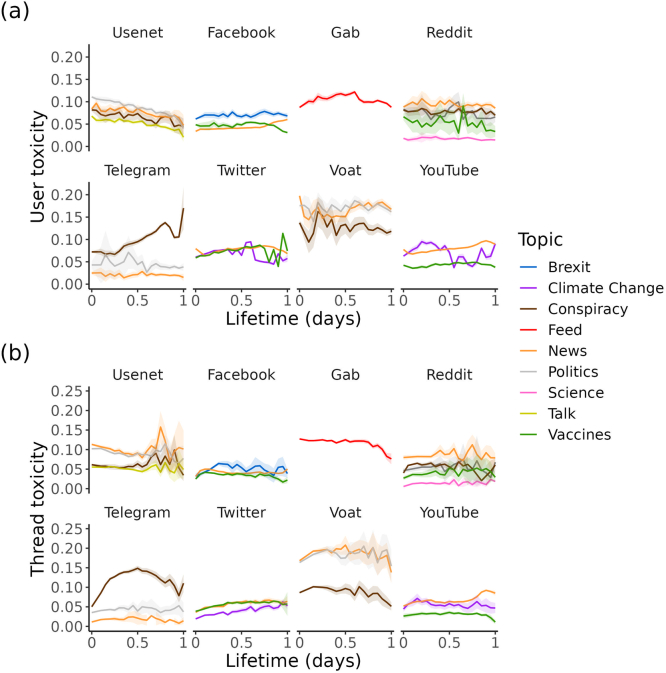
Toxicity is not associated with conversation lifetime. Mean toxicity of **a**. users versus their time of permanence in the dataset and **b**. threads versus their time duration. Trends are reported with their 95% confidence interval and they are reported using a normalized log-binning. Colour-coded legend on the side.

**Extended Data Fig. 5:**
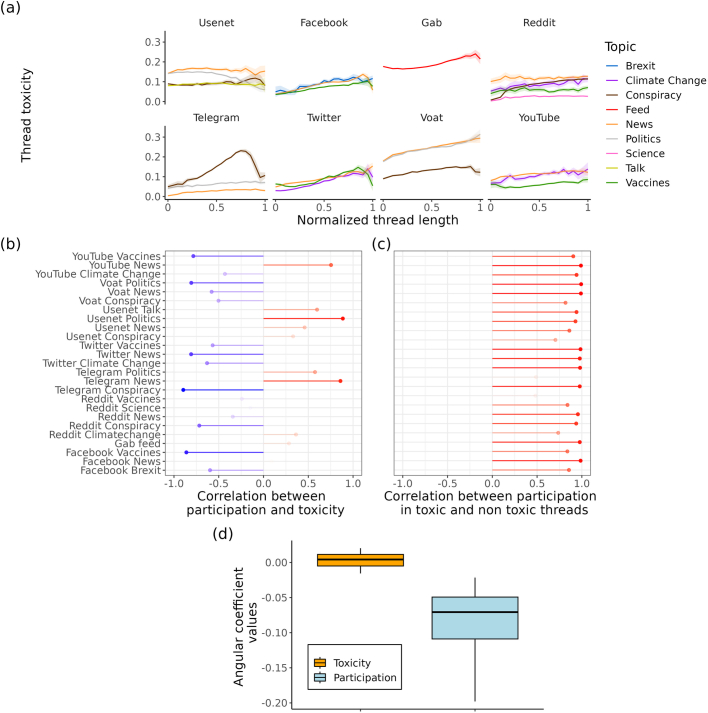
Results hold for a different toxicity threshold. Core analyses presented in the paper repeated employing a lower (0.5) toxicity binary classification threshold. **a**. Mean fraction of toxic comments in conversations versus conversation size, for each dataset (see Fig. 2). Trends are reported with their 95% confidence interval. **b**. Pearson’s correlation coefficients between user participation and toxicity trends for each dataset.**c**. Pearson’s correlation coefficients between users’ participation in toxic and non-toxic thread sets, for each dataset. **d**. Boxplot of the distribution of toxicity (*n* = 25, min = −0.016, max = 0.020, lower whisker = −0.005, *Q*1 = − 0.005, *Q*_2_ = 0.004, *Q*_3_ = 0.012, upper whisker = 0.020) and participation (*n* = 25, min = −0.198, max = −0.022, lower whisker = −0.198, *Q*1 = − 0.109, *Q*_2_ = − 0.070, *Q*_3_ = − 0.049, upper whisker = −0.022) trend slopes for all datasets, as resulting from linear regression. The results of the relative Mann-Kendall tests for trend assessment are shown in Extended Data Table 5.

In the previous sections, we analysed the toxicity level of online conversations after their conclusion. We next focus on how toxicity evolves during a conversation and its effect on the dynamics of the discussion. The common beliefs that (1) online interactions inevitably devolve into toxic exchanges over time and (2) once a conversation reaches a certain toxicity threshold, it would naturally conclude, are not modern notions but they were also prevalent in the early days of the World Wide Web^41^. Assumption 2 aligns with the Perspective API’s definition of toxic language, suggesting that increased toxicity reduces the likelihood of continued participation in a conversation. However, this observation should be reconsidered, as it is not only the peak levels of toxicity that might influence a conversation but, for example, also a consistent rate of toxic content. To test these common assumptions, we used a method similar to that used for measuring participation; we select sufficiently long threads, divide each of them into a fixed number of equal intervals, compute the fraction of toxic comments for each of these intervals, average it over all threads and plot the toxicity trend through the unfolding of the conversations. We find that the average toxicity level remains mostly stable throughout, without showing a distinctive increase around the final part of threads (Fig. 3a (bottom) and Extended Data Fig. 3). Note that a similar observation was made previously^41^, but referring only to Reddit. Our findings challenge the assumption that toxicity discourages people from participating in a conversation, even though this notion is part of the definition of toxicity used by the detection tool. This can be seen by checking the relationship between trends in user participation, a quantity related to the number of users in a discussion at some point, and toxicity. The fact that the former typically decreases while the latter remains stable during conversations indicates that toxicity is not associated with participation in conversations (an example is shown in Fig. 3a; box plots of the slopes of participation and toxicity for the whole dataset are shown in Fig. 3b). This suggests that, on average, people may leave discussions regardless of the toxicity of the exchanges. We calculated the Pearson’s correlation between user participation and toxicity trends for each dataset to support this hypothesis. As shown in Fig. 3d, the resulting correlation coefficients are very heterogeneous, indicating no consistent pattern across different datasets. To further validate this analysis, we tested the differences in the participation of users commenting on either toxic or non-toxic conversations. To split such conversations into two disjoint sets, we first compute the toxicity distribution *T*_*i*_ of long threads in each dataset *i*, and we then label a conversation *j* in dataset *i* as toxic if it has toxicity *t*_*ij*_ ≥ *µ*(*T*_*i*_) + *σ*(*T*_*i*_), with *µ*(*T*_*i*_) being mean and *σ*(*T*_*i*_) the standard deviation of *T*_*i*_; all of the other conversations are considered to be non-toxic. After splitting the threads, for each dataset, we compute the Pearson’s correlation of user participation between sets to find strongly positive values of the coefficient in all cases (Fig. 3c,e). This result is also confirmed by a different analysis of which the results are reported in Supplementary Table 8, in which no significant difference between slopes in toxic and non-toxic threads can be found. Thus, user behaviour in toxic and non-toxic conversations shows almost identical patterns in terms of participation. This reinforces our finding that toxicity, on average, does not appear to affect the likelihood of people participating in a conversation. These analyses were repeated with a lower toxicity classification threshold (Extended Data Fig. 5) and on additional datasets (Supplementary Fig. 2 and Supplementary Table 11), finding consistent results.

## Controversy and toxicity

In this section, we aim to explore why people participate in toxic online conversations and why longer discussions tend to be more toxic. Several factors could be the subject matter. First, controversial topics might lead to longer, more heated debates with increased toxicity. Second, the endorsement of toxic content by other users may act as an incentive to increase the discussion’s toxicity. Third, engagement peaks, due to factors such as reduced discussion focus or the intervention of trolls, may bring a higher share of toxic exchanges. Pursuing this line of inquiry, we identified proxies to measure the level of controversy in conversations and examined how these relate to toxicity and conversation size. Concurrently, we investigated the relationship between toxicity, endorsement and engagement.

**Fig. 4:**
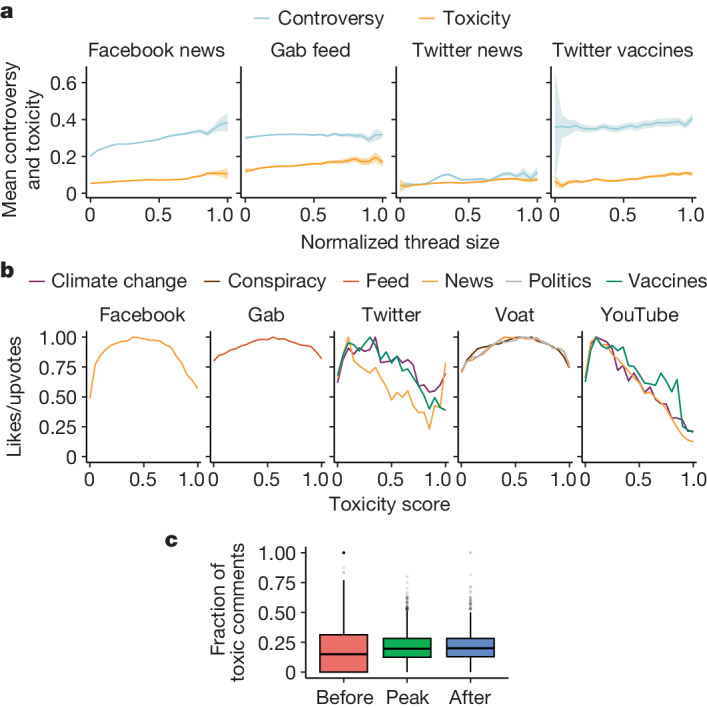
Controversy and toxicity in conversations. **a**, The mean controversy (*σ*(*l*)) and mean toxicity versus thread size (log-binned and normalized) for the Facebook news, Twitter news, Twitter vaccines and Gab feed datasets. Here toxicity is calculated in the same conversations in which controversy could be computed (Extended Data Table 3); the relative Pearson’s, Spearman’s and Kendall’s correlation coefficients are also provided in Extended Data Table 3. Trends are reported with their 95% confidence interval. **b**, Likes/upvotes versus toxicity (linearly binned). **c**, An example (Voat politics dataset) of the distributions of the frequency of toxic comments in threads before (*n* = 2,201, minimum = 0, maximum = 1, lower whisker = 0, Q1 = 0, Q2 = 0.15, Q3 = 0.313, upper whisker = 0.769) at the peak (*n* = 2,798, minimum = 0, maximum = 0.8, lower whisker = 0, Q1 = 0.125, Q2 = 0.196, Q3 = 0.282, upper whisker = 0.513) and after the peak (*n* = 2,791, minimum = 0, maximum = 1, lower whisker = 0, Q1 = 0.129, Q2 = 0.200, Q3 = 0.282, upper whisker = 0.500) of activity, as detected by Kleinberg’s burst detection algorithm.

As shown previously^24,42^, controversy is likely to emerge when people with opposing views engage in the same debate. Thus, the presence of users with diverse political leanings within a conversation could be a valid proxy for measuring controversy. We operationalize this definition as follows. Exploiting the peculiarities of our data, we can infer the political leaning of a subset of users in the Facebook News, Twitter News, Twitter Vaccines and Gab Feed datasets. This is achieved by examining the endorsement, for example, in the form of likes, expressed towards news outlets of which the political inclinations have been independently assessed by news rating agencies (see the ‘Polarization and user leaning attribution’ section of the Methods). Extended Data Table 3 shows a breakdown of the datasets. As a result, we label users with a leaning score *l* ∈ [−1, 1], −1 being left leaning and +1 being right leaning. We then select threads with at least ten different labelled users, in which at least 10% of comments (with a minimum of 20) are produced by such users and assign to each of these comments the same leaning score of those who posted them. In this setting, the level of controversy within a conversation is assumed to be captured by the spread of the political leaning of the participants in the conversation. A natural way for measuring such a spread is the s.d. *σ*(*l*) of the distribution of comments possessing a leaning score: the higher the *σ*(*l*), the greater the level of ideological disagreement and therefore controversy in a thread. We analysed the relationship between controversy and toxicity in online conversations of different sizes. Figure 4a shows that controversy increases with the size of conversations in all datasets, and its trends are positively correlated with the corresponding trends in toxicity (Extended Data Table 3). This supports our hypothesis that controversy and toxicity are closely related in online discussions.

As a complementary analysis, we draw on previous results^43^. In that study, using a definition of controversy operationally different but conceptually related to ours, a link was found between a greater degree of controversy of a discussion topic and a wider distribution of sentiment scores attributed to the set of its posts and comments. We quantified the sentiment of comments using a pretrained BERT model available from Hugging Face^44^, used also in previous studies^45^. The model predicts the sentiment of a sentence through a scoring system ranging from 1 (negative) to 5 (positive). We define the sentiment attributed to a comment *c* as its weighted mean $s(c)=\sum _{i=1.5}{x}_{i}{p}_{i}$, where *x*_*i*_ ∈ [1, 5] is the output score from the model and *p*_*i*_ is the probability associated to that value. Moreover, we normalize the sentiment score *s* for each dataset between 0 and 1. We observe the trends of the mean s.d. of sentiment in conversations, $\bar{\sigma }(s)$, and toxicity are positively correlated for moderated platforms such as Facebook and Twitter but are negatively correlated on Gab (Extended Data Table 3). The positive correlation observed in Facebook and Twitter indicates that greater discrepancies in sentiment of the conversations can, in general, be linked to toxic conversations and vice versa. Instead, on unregulated platforms such as Gab, highly conflicting sentiments seem to be more likely to emerge in less toxic conversations.

As anticipated, another factor that may be associated with the emergence of toxic comments is the endorsement they receive. Indeed, such positive reactions may motivate posting even more comments of the same kind. Using the mean number of likes/upvotes as a proxy of endorsement, we have an indication that this may not be the case. Figure 4b shows that the trend in likes/upvotes versus comments toxicity is never increasing past the toxicity score threshold (0.6).

Finally, to complement our analysis, we inspect the relationship between toxicity and user engagement within conversations, measured as the intensity of the number of comments over time. To do so, we used a method for burst detection^46^ that, after reconstructing the density profile of a temporal stream of elements, separates the stream into different levels of intensity and assigns each element to the level to which it belongs (see the ‘Burst analysis’ section of the Methods). We computed the fraction of toxic comments at the highest intensity level of each conversation and for the levels right before and after it. By comparing the distributions of the fraction of toxic comments for the three intervals, we find that these distributions are statistically different in almost all cases (Fig. 4c and Extended Data Table 4). In all datasets but one, distributions are consistently shifted towards higher toxicity at the peak of engagement, compared with the previous phase. Likewise, in most cases, the peak shows higher toxicity even if compared to the following phase, which in turn is mainly more toxic than the phase before the peak. These results suggest that toxicity is likely to increase together with user engagement.

## Discussion

Here we examine one of the most prominent and persistent characteristics online discussions—toxic behaviour, defined here as rude, disrespectful or unreasonable conduct. Our analysis suggests that toxicity is neither a deterrent to user involvement nor an engagement amplifier; rather, it tends to emerge when exchanges become more frequent and may be a product of opinion polarization. Our findings suggest that the polarization of user opinions—intended as the degree of opposed partisanship of users in a conversation—may have a more crucial role than toxicity in shaping the evolution of online discussions. Thus, monitoring polarization could indicate early interventions in online discussions. However, it is important to acknowledge that the dynamics at play in shaping online discourse are probably multifaceted and require a nuanced approach for effective moderation. Other factors may influence toxicity and engagement, such as the specific subject of the conversation, the presence of influential users or ‘trolls’, the time and day of posting, as well as cultural or demographic aspects, such as user average age or geographical location. Furthermore, even though extremely toxic users are rare (Extended Data Fig. 2), the relationship between participation and toxicity of a discussion may in principle be affected also by small groups of highly toxic and engaged users driving the conversation dynamics. Although the analysis of such subtler aspects is beyond the scope of this Article, they are certainly worth investigating in future research.

However, when people encounter views that contradict their own, they may react with hostility and contempt, consistent with previous research^47^. In turn, it may create a cycle of negative emotions and behaviours that fuels toxicity. We also show that some online conversation features have remained consistent over the past three decades despite the evolution of platforms and social norms.

Our study has some limitations that we acknowledge and discuss. First, we use political leaning as a proxy for general leaning, which may capture only some of the nuances of online opinions. However, political leaning represents a broad spectrum of opinions across different topics, and it correlates well with other dimensions of leaning, such as news preferences, vaccine attitudes and stance on climate change^48,49^. We could not assign a political leaning to users to analyse controversies on all platforms. Still, those considered—Facebook, Gab and Twitter—represent different populations and moderation policies, and the combined data account for nearly 90% of the content in our entire dataset. Our analysis approach is based on breadth and heterogeneity. As such, it may raise concerns about potential reductionism due to the comparison of different datasets from different sources and time periods. We acknowledge that each discussion thread, platform and context has unique characteristics and complexities that might be diminished when homogenizing data. However, we aim not to capture the full depth of every discussion but to identify and highlight general patterns and trends in online toxicity across platforms and time. The quantitative approach used in our study is similar to numerous other studies^15^ and enables us to uncover these overarching principles and patterns that may otherwise remain hidden. Of course, it is not possible to account for the behaviours of passive users. This entails, for example, that even if toxicity does not seem to make people leave conversations, it could still be a factor that discourages them from joining them. Our study leverages an extensive dataset to examine the intricate relationship between persistent online human behaviours and the characteristics of different social media platforms. Our findings challenge the prevailing assumption by demonstrating that toxic content, as traditionally defined, does not necessarily reduce user engagement, thereby questioning the assumed direct correlation between toxic content and negative discourse dynamics. This highlights the necessity for a detailed examination of the effect of toxic interactions on user behaviour and the quality of discussions across various platforms. Our results, showing user resilience to toxic content, indicate the potential for creating advanced, context-aware moderation tools that can accurately navigate the complex influence of antagonistic interactions on community engagement and discussion quality. Moreover, our study sets the stage for further exploration into the complexities of toxicity and its effect on engagement within online communities. Advancing our grasp of online discourse necessitates refining content moderation techniques grounded in a thorough understanding of human behaviour. Thus, our research adds to the dialogue on creating more constructive online spaces, promoting moderation approaches that are effective yet nuanced, facilitating engaging exchanges and reducing the tangible negative effects of toxic behaviour.

Through the extensive dataset presented here, critical aspects of the online platform ecosystem and fundamental dynamics of user interactions can be explored. Moreover, we provide insights that a comparative approach such as the one followed here can prove invaluable in discerning human behaviour from platform-specific features. This may be used to investigate further sensitive issues, such as the formation of polarization and misinformation. The resulting outcomes have multiple potential impacts. Our findings reveal consistent toxicity patterns across platforms, topics and time, suggesting that future research in this field should prioritize the concept of invariance. Recognizing that toxic behaviour is a widespread phenomenon that is not limited by platform-specific features underscores the need for a broader, unified approach to understanding online discourse. Furthermore, the participation of users in toxic conversations suggests that a simple approach to removing toxic comments may not be sufficient to prevent user exposure to such phenomena. This indicates a need for more sophisticated moderation techniques to manage conversation dynamics, including early interventions in discussions that show warnings of becoming toxic. Furthermore, our findings support the idea that examining content pieces in connection with others could enhance the effectiveness of automatic toxicity detection models. The observed homogeneity suggests that models trained using data from one platform may also have applicability to other platforms. Future research could explore further into the role of controversy and its interaction with other elements contributing to toxicity. Moreover, comparing platforms could enhance our understanding of invariant human factors related to polarization, disinformation and content consumption. Such studies would be instrumental in capturing the drivers of the effect of social media platforms on human behaviour, offering valuable insights into the underlying dynamics of online interactions.

## Methods

### Data collection

In our study, data collection from various social media platforms was strategically designed to encompass various topics, ensuring maximal heterogeneity in the discussion themes. For each platform, where feasible, we focus on gathering posts related to diverse areas such as politics, news, environment and vaccinations. This approach aims to capture a broad spectrum of discourse, providing a comprehensive view of conversation dynamics across different content categories.

#### Facebook

We use datasets from previous studies that covered discussions about vaccines^50^, news^51^ and brexit^52^. For the vaccines topic, the resulting dataset contains around 2 million comments retrieved from public groups and pages in a period that ranges from 2 January 2010 to 17 July 2017. For the news topic, we selected a list of pages from the Europe Media Monitor that reported the news in English. As a result, the obtained dataset contains around 362 million comments between 9 September 2009 and 18 August 2016. Furthermore, we collect a total of about 4.5 billion likes that the users put on posts and comments concerning these pages. Finally, for the brexit topic, the dataset contains around 460,000 comments from 31 December 2015 to 29 July 2016.

#### Gab

We collect data from the Pushshift.io archive (https://files.pushshift.io/gab/) concerning discussions taking place from 10 August 2016, when the platform was launched, to 29 October 2018, when Gab went temporarily offline due to the Pittsburgh shooting^53^. As a result, we collect a total of around 14 million comments.

#### Reddit

Data were collected from the Pushshift.io archive (https://pushshift.io/) for the period ranging from 1 January 2018 to 31 December 2022. For each topic, whenever possible, we manually identified and selected subreddits that best represented the targeted topics. As a result of this operation, we obtained about 800,000 comments from the r/conspiracy subreddit for the conspiracy topic. For the vaccines topic, we collected about 70,000 comments from the r/VaccineDebate subreddit, focusing on the COVID-19 vaccine debate. We collected around 400,000 comments from the r/News subreddit for the news topic. We collected about 70,000 comments from the r/environment subreddit for the climate change topic. Finally, we collected around 550,000 comments from the r/science subreddit for the science topic.

#### Telegram

We created a list of 14 channels, associating each with one of the topics considered in the study. For each channel, we manually collected messages and their related comments. As a result, from the four channels associated with the news topic (news notiziae, news ultimora, news edizionestraordinaria, news covidultimora), we obtained around 724,000 comments from posts between 9 April 2018 and 20 December 2022. For the politics topic, instead, the corresponding two channels (politics besttimeline, politics polmemes) produced a total of around 490,000 comments between 4 August 2017 and 19 December 2022. Finally, the eight channels assigned to the conspiracy topic (conspiracy bennyjhonson, conspiracy tommyrobinsonnews, conspiracy britainsfirst, conspiracy loomeredofficial, conspiracy thetrumpistgroup, conspiracy trumpjr, conspiracy pauljwatson, conspiracy iononmivaccino) produced a total of about 1.4 million comments between 30 August 2019 and 20 December 2022.

#### Twitter

We used a list of datasets from previous studies that includes discussions about vaccines^54^, climate change^49^ and news^55^ topics. For the vaccines topic, we collected around 50 million comments from 23 January 2010 to 25 January 2023. For the news topic, we extend the dataset used previously^55^ by collecting all threads composed of less than 20 comments, obtaining a total of about 9.5 million comments for a period ranging from 1 January 2020 to 29 November 2022. Finally, for the climate change topic, we collected around 9.7 million comments between 1 January 2020 and 10 January 2023.

#### Usenet

We collected data for the Usenet discussion system by querying the Usenet Archive (https://archive.org/details/usenet?tab=about). We selected a list of topics considered adequate to contain a large, broad and heterogeneous number of discussions involving active and populated newsgroups. As a result of this selection, we selected conspiracy, politics, news and talk as topic candidates for our analysis. For the conspiracy topic, we collected around 280,000 comments between 1 September 1994 and 30 December 2005 from the alt.conspiracy newsgroup. For the politics topics, we collected around 2.6 million comments between 29 June 1992 and 31 December 2005 from the alt.politics newsgroup. For the news topic, we collected about 620,000 comments between 5 December 1992 and 31 December 2005 from the alt.news newsgroup. Finally, for the talk topic, we collected all of the conversations from the homonym newsgroup on a period that ranges from 13 February 1989 to 31 December 2005 for around 2.1 million contents.

#### Voat

We used a dataset presented previously^56^ that covers the entire lifetime of the platform, from 9 January 2018 to 25 December 2020, including a total of around 16.2 million posts and comments shared by around 113,000 users in about 7,100 subverses (the equivalent of a subreddit for Voat). Similarly to previous platforms, we associated the topics to specific subverses. As a result of this operation, for the conspiracy topic, we collected about 1 million comments from the greatawakening subverse between 9 January 2018 and 25 December 2020. For the politics topic, we collected around 1 million comments from the politics subverse between 16 June 2014 and 25 December 2020. Finally, for the news topic, we collected about 1.4 million comments from the news subverse between 21 November 2013 and 25 December 2020.

#### YouTube

We used a dataset proposed in previous studies that collected conversations about the climate change topic^49^, which is extended, coherently with previous platforms, by including conversations about vaccines and news topics. The data collection process for YouTube is performed using the YouTube Data API (https://developers.google.com/youtube/v3). For the climate change topic, we collected around 840,000 comments between 16 March 2014 and 28 February 2022. For the vaccines topic, we collected conversations between 31 January 2020 and 24 October 2021 containing keywords about COVID-19 vaccines, namely Sinopharm, CanSino, Janssen, Johnson&Johnson, Novavax, CureVac, Pfizer, BioNTech, AstraZeneca and Moderna. As a result of this operation, we gathered a total of around 2.6 million comments to videos. Finally, for the news topic, we collected about 20 million comments between 13 February 2006 and 8 February 2022, including videos and comments from a list of news outlets, limited to the UK and provided by Newsguard (see the ‘Polarization and user leaning attribution’ section).

### Content moderation policies

Content moderation policies are guidelines that online platforms use to monitor the content that users post on their sites. Platforms have different goals and audiences, and their moderation policies may vary greatly, with some placing more emphasis on free expression and others prioritizing safety and community guidelines.

Facebook and YouTube have strict moderation policies prohibiting hate speech, violence and harassment^57^. To address harmful content, Facebook follows a ‘remove, reduce, inform’ strategy and uses a combination of human reviewers and artificial intelligence to enforce its policies^58^. Similarly, YouTube has a similar set of community guidelines regarding hate speech policy, covering a wide range of behaviours such as vulgar language^59^, harassment^60^ and, in general, does not allow the presence of hate speech and violence against individuals or groups based on various attributes^61^. To ensure that these guidelines are respected, the platform uses a mix of artificial intelligence algorithms and human reviewers^62^.

Twitter also has a comprehensive content moderation policy and specific rules against hateful conduct^63,64^. They use automation^65^ and human review in the moderation process^66^. At the date of submission, Twitter’s content policies have remained unchanged since Elon Musk’s takeover, except that they ceased enforcing their COVID-19 misleading information policy on 23 November 2022. Their policy enforcement has faced criticism for inconsistency^67^.

Reddit falls somewhere in between regarding how strict its moderation policy is. Reddit’s content policy has eight rules, including prohibiting violence, harassment and promoting hate based on identity or vulnerability^68,69^. Reddit relies heavily on user reports and volunteer moderators. Thus, it could be considered more lenient than Facebook, YouTube and Twitter regarding enforcing rules. In October 2022, Reddit announced that they intend to update their enforcement practices to apply automation in content moderation^70^.

By contrast, Telegram, Gab and Voat take a more hands-off approach with fewer restrictions on content. Telegram has ambiguity in its guidelines, which arises from broad or subjective terms and can lead to different interpretations^71^. Although they mentioned they may use automated algorithms to analyse messages, Telegram relies mainly on users to report a range of content, such as violence, child abuse, spam, illegal drugs, personal details and pornography^72^. According to Telegram’s privacy policy, reported content may be checked by moderators and, if it is confirmed to violate their terms, temporary or permanent restrictions may be imposed on the account^73^. Gab’s Terms of Service allow all speech protected under the First Amendment to the US Constitution, and unlawful content is removed. They state that they do not review material before it is posted on their website and cannot guarantee prompt removal of illegal content after it has been posted^74^. Voat was once known as a ‘free-speech’ alternative to Reddit and allowed content even if it may be considered offensive or controversial^56^.

Usenet is a decentralized online discussion system created in 1979. Owing to its decentralized nature, Usenet has been difficult to moderate effectively, and it has a reputation for being a place where controversial and even illegal content can be posted without consequence. Each individual group on Usenet can have its own moderators, who are responsible for monitoring and enforcing their group’s rules, and there is no single set of rules that applies to the entire platform^75^.

### Logarithmic binning and conversation size

Owing to the heavy-tailed distributions of conversation length (Extended Data Fig. 1), to plot the figures and perform the analyses, we used logarithmic binning. Thus, according to its length, each thread of each dataset is assigned to 1 out of 21 bins. To ensure a minimal number of points in each bin, we iteratively change the left bound of the last bin so that it contains at least *N* = 50 elements (we set *N* = 100 in the case of Facebook news, due to its larger size). Specifically, considering threads ordered in increasing length, the size of the largest thread is changed to that of the second last largest one, and the binning is recalculated accordingly until the last bin contains at least *N* points.

For visualization purposes, we provide a normalization of the logarithmic binning outcome that consists of mapping discrete points into coordinates of the *x* axis such that the bins correspond to {0, 0.05, 0.1, ..., 0.95, 1}.

To perform the part of the analysis, we select conversations belonging to the [0.7, 1] interval of the normalized logarithmic binning of thread length. This interval ensures that the conversations are sufficiently long and that we have a substantial number of threads. Participation and toxicity trends are obtained by applying to such conversations a linear binning of 21 elements to a chronologically ordered sequence of comments, that is, threads. A breakdown of the resulting datasets is provided in Supplementary Table 2.

Finally, to assess the equality of the growth rates of participation values in toxic and non-toxic threads (see the ‘Conversation evolution and toxicity’ section), we implemented the following linear regression model:

$${\rm{p}}{\rm{a}}{\rm{r}}{\rm{t}}{\rm{i}}{\rm{c}}{\rm{i}}{\rm{p}}{\rm{a}}{\rm{t}}{\rm{i}}{\rm{o}}{\rm{n}}={\beta }_{0}+{\beta }_{1}\cdot {\rm{b}}{\rm{i}}{\rm{n}}+{\beta }_{2}\cdot \,({\rm{b}}{\rm{i}}{\rm{n}}\cdot {\rm{i}}{\rm{s}}{\rm{T}}{\rm{o}}{\rm{x}}{\rm{i}}{\rm{c}}),$$

where the term *β*_2_ accounts for the effect that being a toxic conversation has on the growth of participation. Our results show that *β*_2_ is not significantly different from 0 in most original and validation datasets (Supplementary Tables 8 and 11)

### Toxicity detection and validation of the models used

The problem of detecting toxicity is highly debated, to the point that there is currently no agreement on the very definition of toxic speech^64,76^. A toxic comment can be regarded as one that includes obscene or derogatory language^32^, that uses harsh, abusive language and personal attacks^33^, or contains extremism, violence and harassment^11^, just to give a few examples. Even though toxic speech should, in principle, be distinguished from hate speech, which is commonly more related to targeted attacks that denigrate a person or a group on the basis of attributes such as race, religion, gender, sex, sexual orientation and so on^77^, it sometimes may also be used as an umbrella term^78,79^. This lack of agreement directly reflects the challenging and inherent subjective nature of the concept of toxicity. The complexity of the topic makes it particularly difficult to assess the reliability of natural language processing models for automatic toxicity detection despite the impressive improvements in the field. Modern natural language processing models, such as Perspective API, are deep learning models that leverage word-embedding techniques to build representations of words as vectors in a high-dimensional space, in which a metric distance should reflect the conceptual distance among words, therefore providing linguistic context. A primary concern regarding toxicity detection models is their limited ability to contextualize conversations^11,80^. These models often struggle to incorporate factors beyond the text itself, such as the participant’s personal characteristics, motivations, relationships, group memberships and the overall tone of the discussion^11^. Consequently, what is considered to be toxic content can vary significantly among different groups, such as ethnicities or age groups^81^, leading to potential biases. These biases may stem from the annotators’ backgrounds and the datasets used for training, which might not adequately represent cultural heterogeneity. Moreover, subtle forms of toxic content, like indirect allusions, memes and inside jokes targeted at specific groups, can be particularly challenging to detect. Word embeddings equip current classifiers with a rich linguistic context, enhancing their ability to recognize a wide range of patterns characteristic of toxic expression. However, the requirements for understanding the broader context of a conversation, such as personal characteristics, motivations and group dynamics, remain beyond the scope of automatic detection models. We acknowledge these inherent limitations in our approach. Nonetheless, reliance on automatic detection models is essential for large-scale analyses of online toxicity like the one conducted in this study. We specifically resort to the Perspective API for this task, as it represents state-of-the-art automatic toxicity detection, offering a balance between linguistic nuance and scalable analysis capabilities. To define an appropriate classification threshold, we draw from the existing literature^64^, which uses 0.6 as the threshold for considering a comment to be toxic. This threshold can also be considered a reasonable one as, according to the developer guidelines offered by Perspective, it would indicate that the majority of the sample of readers, namely 6 out of 10, would perceive that comment as toxic. Due to the limitations mentioned above (for a criticism of Perspective API, see ref. ^82^), we validate our results by performing a comparative analysis using two other toxicity detectors: Detoxify (https://github.com/unitaryai/detoxify), which is similar to Perspective, and IMSYPP, a classifier developed for a European Project on hate speech^16^ (https://huggingface.co/IMSyPP). In Supplementary Table 14, the percentages of agreement among the three models in classifying 100,000 comments taken randomly from each of our datasets are reported. For Detoxify we used the same binary toxicity threshold (0.6) as used with Perspective. Although IMSYPP operates on a distinct definition of toxicity as outlined previously^16^, our comparative analysis shows a general agreement in the results. This alignment, despite the differences in underlying definitions and methodologies, underscores the robustness of our findings across various toxicity detection frameworks. Moreover, we perform the core analyses of this study using all classifiers on a further, vast and heterogeneous dataset. As shown in Supplementary Figs. 1 and 2, the results regarding toxicity increase with conversation size and user participation and toxicity are quantitatively very similar. Furthermore, we verify the stability of our findings under different toxicity thresholds. Although the main analyses in this paper use the threshold value recommended by the Perspective API, set at 0.6, to minimize false positives, our results remain consistent even when applying a less conservative threshold of 0.5. This is demonstrated in Extended Data Fig. 5, confirming the robustness of our observations across varying toxicity levels. For this study, we used the API support for languages prevalent in the European and American continents, including English, Spanish, French, Portuguese, German, Italian, Dutch, Polish, Swedish and Russian. Detoxify also offers multilingual support. However, IMSYPP is limited to English and Italian text, a factor considered in our comparative analysis.

### Polarization and user leaning attribution

Our approach to measuring controversy in a conversation is based on estimating the degree of political partisanship among the participants. This measure is closely related to the political science concept of political polarization. Political polarization is the process by which political attitudes diverge from moderate positions and gravitate towards ideological extremes, as described previously^83^. By quantifying the level of partisanship within discussions, we aim to provide insights into the extent and nature of polarization in online debates. In this context, it is important to distinguish between ‘ideological polarization’ and ‘affective polarization’. Ideological polarization refers to divisions based on political viewpoints. By contrast, affective polarization is characterized by positive emotions towards members of one’s group and hostility towards those of opposing groups^84,85^. Here we focus specifically on ideological polarization. The subsequent description of our procedure for attributing user political leanings will further clarify this focus. On online social media, the individual leaning of a user toward a topic can be inferred through the content produced or the endorsement shown toward specific content. In this study, we consider the endorsement of users to news outlets of which the political leaning has been evaluated by trustworthy external sources. Although not without limitations—which we address below—this is a standard approach that has been used in several studies, and has become a common and established practice in the field of social media analysis due to its practicality and effectiveness in providing a broad understanding of political dynamics on these online platforms^1,43,86–88^. We label news outlets with a political score based on the information reported by Media Bias/Fact Check (MBFC) (https://mediabiasfactcheck.com), integrating with the equivalent information from Newsguard (https://www.newsguardtech.com/). MBFC is an independent fact-checking organization that rates news outlets on the basis of the reliability and the political bias of the content that they produce and share. Similarly, Newsguard is a tool created by an international team of journalists that provides news outlet trust and political bias scores. Following standard methods used in the literature^1,43^, we calculated the individual leaning of a user *l* ∈ [−1, 1] as the average of the leaning scores *l*_*c*_ ∈ [−1, 1] attributed to each of the content it produced/shared, where *l*_*c*_ results from a mapping of the news organizations political scores provided by MBFC and Newsguard, respectively: [left, centre-left, centre, centre-right, right] to [−1, − 0.5, 0, 0.5, 1], and [far left, left, right, far right] to [−1, −0.5, 0.5, 1]). Our datasets have different structures, so we have to evaluate user leanings in different ways. For Facebook News, we assign a leaning score to users who posted a like at least three times and commented at least three times under news outlet pages that have a political score. For Twitter News, a leaning is assigned to users who posted at least 15 comments under scored news outlet pages. For Twitter Vaccines and Gab, we consider users who shared content produced by scored news outlet pages at least three times. A limitation of our approach is that engaging with politically aligned content does not always imply agreement; users may interact with opposing viewpoints for critical discussion. However, research indicates that users predominantly share content aligning with their own views, especially in politically charged contexts^87,89,90^. Moreover, our method captures users who actively express their political leanings, omitting the ‘passive’ ones. This is due to the lack of available data on users who do not explicitly state their opinions. Nevertheless, analysing active users offers valuable insights into the discourse of those most engaged and influential on social media platforms.

### Burst analysis

We used the Kleinberg burst detection algorithm^46^ (see the ‘Controversy and toxicity’ section) to all conversations with at least 50 comments in a dataset. In our analysis, we randomly sample up to 5,000 conversations, each containing a specific number of comments. To ensure the reliability of our data, we exclude conversations with an excessive number of double timestamps—defined as more than 10 consecutive or over 100 within the first 24 h. This criterion helps to mitigate the influence of bots, which could distort the patterns of human activity. Furthermore, we focus on the first 24 h of each thread to analyse streams of comments during their peak activity period. Consequently, Usenet was excluded from our study. The unique usage characteristics of Usenet render such a time-constrained analysis inappropriate, as its activity patterns do not align with those of the other platforms under consideration. By reconstructing the density profile of the comment stream, the algorithm divides the entire stream’s interval into subintervals on the basis of their level of intensity. Labelled as discrete positive values, higher levels of burstiness represent higher activity segments. To avoid considering flat-density phases, threads with a maximum burst level equal to 2 are excluded from this analysis. To assess whether a higher intensity of comments results in a higher comment toxicity, we perform a Mann–Whitney *U*-test^91^ with Bonferroni correction for multiple testing between the distributions of the fraction of toxic comments *t*_*i*_ in three intensity phases: during the peak of engagement and at the highest levels before and after. Extended Data Table 4 shows the corrected *P* values of each test, at a 0.99 confidence level, with H1 indicated in the column header. An example of the distribution of the frequency of toxic comments in threads at the three phases of a conversation considered (pre-peak, peak and post-peak) is reported in Fig. 4c.

### Toxicity detection on Usenet

As discussed in the section on toxicity detection and the Perspective API above, automatic detectors derive their understanding of toxicity from the annotated datasets that they are trained on. The Perspective API is predominantly trained on recent texts, and its human labellers conform to contemporary cultural norms. Thus, although our dataset dates back to no more than the early 1990s, we provide a discussion on the viability of the application of Perspective API to Usenet and validation analysis. Contemporary society, especially in Western contexts, is more sensitive to issues of toxicity, including gender, race and sexual orientation, compared with a few decades ago. This means that some comments identified as toxic today, including those from older platforms like Usenet, might not have been considered as such in the past. However, this discrepancy does not significantly affect our analysis, which is centred on current standards of toxicity. On the other hand, changes in linguistic features may have some repercussions: there may be words and locutions that were frequently used in the 1990s that instead appear sparsely in today’s language, making Perspective potentially less effective in classifying short texts that contain them. We therefore proceeded to evaluate the impact that such a possible scenario could have on our results. In light of the above considerations, we consider texts labelled as toxic as correctly classified; instead, we assume that there is a fixed probability *p* that a comment may be incorrectly labelled as non-toxic. Consequently, we randomly designate a proportion *p* of non-toxic comments, relabel them as toxic and compute the toxicity versus conversation size trend (Fig. 2) on the altered dataset across various *p*. Specifically, for each value, we simulate 500 different trends, collecting their regression slopes to obtain a null distribution for them. To assess if the probability of error could lead to significant differences in the observed trend, we compute the fraction *f* of slopes lying outside the interval (−|*s*|,|*s*|), where *s* is the slope of the observed trend. We report the result in Supplementary Table 9 for different values of *p*. In agreement with our previous analysis, we assume that the slope differs significantly from the ones obtained from randomized data if *f* is less than 0.05.

We observed that only the Usenet Talk dataset shows sensitivity to small error probabilities, and the others do not show a significant difference. Consequently, our results indicate that Perspective API is suitable for application to Usenet data in our analyses, notwithstanding the potential linguistic and cultural shifts that might affect the classifier’s reliability with older texts.

### Toxicity of short conversations

Our study focuses on the relationship between user participation and the toxicity of conversations, particularly in engaged or prolonged discussions. A potential concern is that concentrating on longer threads overlooks conversations that terminate quickly due to early toxicity, therefore potentially biasing our analysis. To address this, we analysed shorter conversations, comprising 6 to 20 comments, in each dataset. In particular, we computed the distributions of toxicity scores of the first and last three comments in each thread. This approach helps to ensure that our analysis accounts for a range of conversation lengths and patterns of toxicity development, providing a more comprehensive understanding of the dynamics at play. As shown in Supplementary Fig. 3, for each dataset, the distributions of the toxicity scores display high similarity, meaning that, in short conversations, the last comments are not significantly more toxic than the initial ones, indicating that the potential effects mentioned above do not undermine our conclusions. Regarding our analysis of longer threads, we notice here that the participation quantity can give rise to similar trends in various cases. For example, high participation can be achieved because many users take part in the conversation, but also with small groups of users in which everyone is equally contributing over time. Or, in very large discussions, the contributions of individual outliers may remain hidden. By measuring participation, these and other borderline cases may not be distinct from the statistically highly likely discussion dynamics but, ultimately, this lack of discriminatory power does not have any implications on our findings nor on the validity of the conclusions that we draw.

### Reporting summary

Further information on research design is available in the Nature Portfolio Reporting Summary linked to this article.

## Online content

Any methods, additional references, Nature Portfolio reporting summaries, source data, extended data, supplementary information, acknowledgements, peer review information; details of author contributions and competing interests; and statements of data and code availability are available at 10.1038/s41586-024-07229-y.

## Supplementary information

Supplementary Information Supplementary Information 1–4, including details regarding data collection for validation dataset, Supplementary Figs. 1–3, Supplementary Tables 1–17 and software and coding specifications.

Reporting Summary

## Figures and Tables

**Extended Data Table 1:**
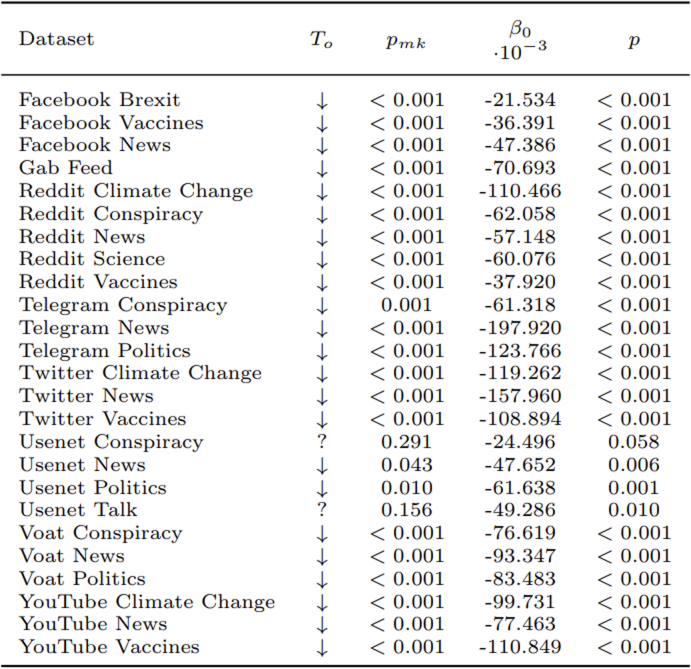
Results of Mann-Kendall tests applied to participation vs normalized comment position

The table contains the resulting trend (*T*_*o*_), *p* value of the test (*p*_*mk*_), slopes from linear regression (*β*_0_) and its *p* value.

**Extended Data Table 2:**
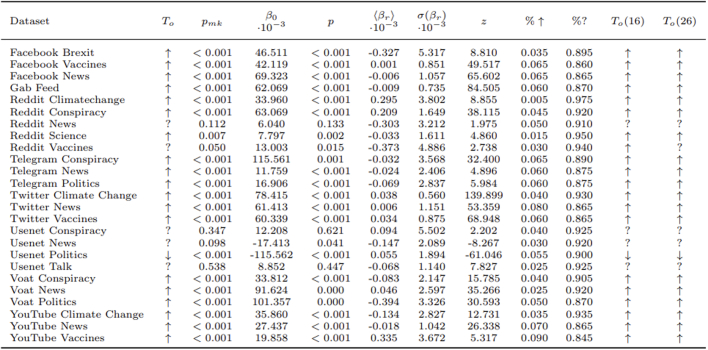
Toxicity versus conversation size

Trend in toxicity versus conversation size *T*_*o*_ as resulting from a Mann-Kendall test. The table shows the *p* value *p*_*mk*_ of the test, the slope of linear regression *β*_0_, its *p*−value, the mean angular coefficient from 200 randomizations of the binary toxicity label ⟨*β*_*r*_⟩, the standard deviation of their resulting distributions *σ*(*β*_*r*_), the *z*−score of the observed value, the percentage of randomizations resulting in an increasing trend % ↑, the percentage of randomizations resulting in an ambiguous trend %?, trend in toxicity for 16 and 26 size intervals *T*_*o*_(16), *T*_*o*_(26). For randomizations and other size intervals, a random subset of the Facebook news dataset containing ∼6.5 M comments was used.

**Extended Data Table 3:**
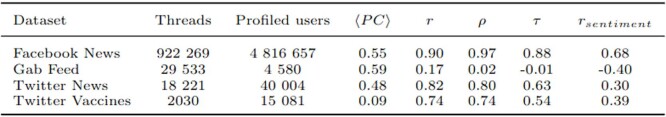
The datasets used in the analysis of controversy

For each dataset, we report the number of conversations (Threads), the number of users to which a political leaning could be assigned (Profiled users), the mean percentage of comments from a profiled user in the conversations (⟨*PC*⟩), the Pearson’s *r*, Spearman’s *ρ* and Kendall’s *τ* correlations between the trends in toxicity and controversy. The *r*_*sentiment*_, instead, represents Pearson’s *r* correlation score between the trends in toxicity and sentiment.

**Extended Data Table 4:**
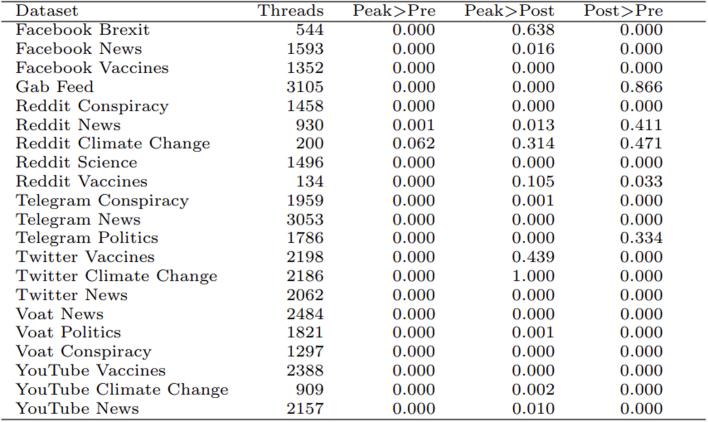
Conversations are more toxic at the peak of activity

Burst analysis of activity in conversations. For each dataset, the number of threads considered in the analysis based on all the criteria listed in Burst Analysis in the Methods, along with the p-values for the hypothesis (H1) that the distributions in toxicity are more skewed towards higher toxicity content at the peak of activity w.r.t. previous and subsequent activity levels (Peak *>* Pre and Peak *>* Post, respectively), and after the peak compared to before the peak (Post *>* Pre). H1 is considered accepted if *p* < 0.01.

**Extended Data Table 5:**
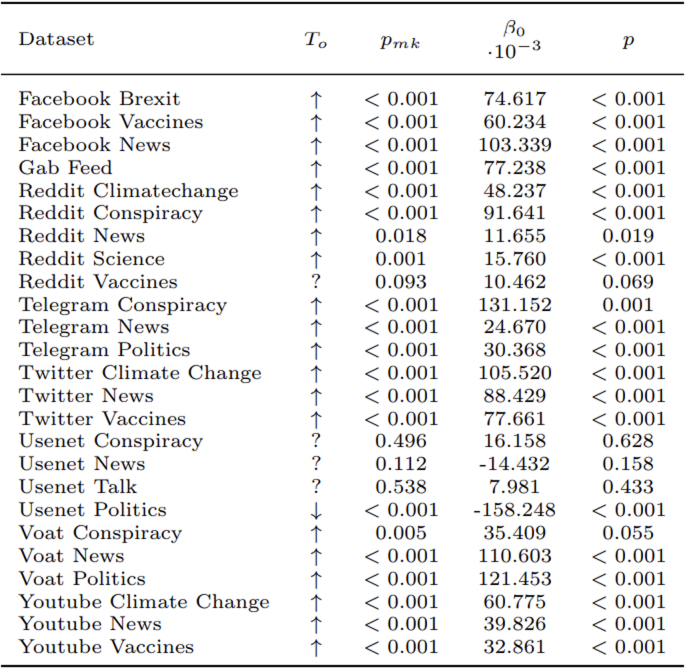
Results of Mann-Kendall tests applied to the toxicity vs conversation size trends

The table shows the detected trend (*T*_*o*_) the *p* value of the test *p*_*mk*_, slopes from linear regression (*β*_0_) and its *p* value using 0.5 as threshold for toxicity.

## Data Availability

Facebook, Twitter and YouTube data are made available in accordance with their respective terms of use. IDs of comments used in this work are provided at Open Science Framework (10.17605/osf.io/fq5dy). For the remaining platforms (Gab, Reddit, Telegram, Usenet and Voat), all of the necessary information to recreate the datasets used in this study can be found in the ‘Data collection’ section.

## Code availability

The code used for the analyses presented in the Article is available at Open Science Framework (10.17605/osf.io/fq5dy). The repository includes dummy datasets to illustrate the required data format and make the code run.

## References

[1] Cinelli, M., Morales, G. D. F., Galeazzi, A., Quattrociocchi, W. & Starnini, M. The echo chamber effect on social media. *Proc. Natl Acad. Sci. USA* **118**, e2023301118 (2021). 33622786 10.1073/pnas.2023301118 PMC7936330

[2] Tucker, J. A. et al. Social media, political polarization, and political disinformation: a review of the scientific literature. Preprint at *SSRN*10.2139/ssrn.3144139 (2018).

[3] González-Bailón, S. et al. Asymmetric ideological segregation in exposure to political news on Facebook. *Science* **381**, 392–398 (2023). 37499003 10.1126/science.ade7138

[4] Guess, A. et al. How do social media feed algorithms affect attitudes and behavior in an election campaign? *Science* **381**, 398–404 (2023). 37498999 10.1126/science.abp9364

[5] Del Vicario, M. et al. The spreading of misinformation online. *Proc. Natl Acad. Sci. USA* **113**, 554–559 (2016). 26729863 10.1073/pnas.1517441113 PMC4725489

[6] Bakshy, E., Messing, S. & Adamic, L. A. Exposure to ideologically diverse news and opinion on Facebook. *Science* **348**, 1130–1132 (2015). 25953820 10.1126/science.aaa1160

[7] Bail, C. A. et al. Exposure to opposing views on social media can increase political polarization. *Proc. Natl Acad. Sci. USA* **115**, 9216–9221 (2018). 30154168 10.1073/pnas.1804840115 PMC6140520

[8] Nyhan, B. et al. Like-minded sources on Facebook are prevalent but not polarizing. *Nature* **620**, 137–144 (2023). 37500978 10.1038/s41586-023-06297-w PMC10396953

[9] Guess, A. et al. Reshares on social media amplify political news but do not detectably affect beliefs or opinions. *Science* **381**, 404–408 (2023). 37499012 10.1126/science.add8424

[10] Castaño-Pulgaŕın, S. A., Suárez-Betancur, N., Vega, L. M. T. & López, H. M. H. Internet, social media and online hate speech. Systematic review. *Aggress. Viol. Behav.* **58**, 101608 (2021).

[11] Sheth, A., Shalin, V. L. & Kursuncu, U. Defining and detecting toxicity on social media: context and knowledge are key. *Neurocomputing* **490**, 312–318 (2022).

[12] Lupu, Y. et al. Offline events and online hate. *PLoS ONE* **18**, e0278511 (2023). 36696388 10.1371/journal.pone.0278511 PMC9876356

[13] Gentzkow, M. & Shapiro, J. M. Ideological segregation online and offline. *Q. J. Econ.* **126**, 1799–1839 (2011).

[14] Aichner, T., Grünfelder, M., Maurer, O. & Jegeni, D. Twenty-five years of social media: a review of social media applications and definitions from 1994 to 2019. *Cyberpsychol. Behav. Social Netw.* **24**, 215–222 (2021). 10.1089/cyber.2020.0134 PMC8064945 33847527

[15] Lazer, D. M. et al. The science of fake news. *Science* **359**, 1094–1096 (2018). 29590025 10.1126/science.aao2998

[16] Cinelli, M. et al. Dynamics of online hate and misinformation. *Sci. Rep.* **11**, 22083 (2021). 34764344 10.1038/s41598-021-01487-w PMC8585974

[17] González-Bailón, S. & Lelkes, Y. Do social media undermine social cohesion? A critical review. *Soc. Issues Pol. Rev.* **17**, 155–180 (2023).

[18] Roozenbeek, J. & Zollo, F. Democratize social-media research—with access and funding. *Nature* **612**, 404–404 (2022). 36513834 10.1038/d41586-022-04407-8

[19] Dutton, W. H. Network rules of order: regulating speech in public electronic fora. *Media Cult. Soc.* **18**, 269–290 (1996).

[20] Papacharissi, Z. Democracy online: civility, politeness, and the democratic potential of online political discussion groups. *N. Media Soc.* **6**, 259–283 (2004).

[21] Coe, K., Kenski, K. & Rains, S. A. Online and uncivil? Patterns and determinants of incivility in newspaper website comments. *J. Commun.* **64**, 658–679 (2014).

[22] Anderson, A. A., Brossard, D., Scheufele, D. A., Xenos, M. A. & Ladwig, P. The “nasty effect:” online incivility and risk perceptions of emerging technologies. *J. Comput. Med. Commun.* **19**, 373–387 (2014).

[23] Garrett, R. K. Echo chambers online?: Politically motivated selective exposure among internet news users. *J. Comput. Med. Commun.* **14**, 265–285 (2009).

[24] Del Vicario, M. et al. Echo chambers: emotional contagion and group polarization on Facebook. *Sci. Rep.* **6**, 37825 (2016). 27905402 10.1038/srep37825 PMC5131349

[25] Garimella, K., De Francisci Morales, G., Gionis, A. & Mathioudakis, M. Echo chambers, gatekeepers, and the price of bipartisanship. In *Proc. 2018 World Wide Web Conference*, 913–922 (International World Wide Web Conferences Steering Committee, 2018).

[26] Johnson, N. et al. Hidden resilience and adaptive dynamics of the global online hate ecology. *Nature* **573**, 261–265 (2019). 31435010 10.1038/s41586-019-1494-7

[27] Fortuna, P. & Nunes, S. A survey on automatic detection of hate speech in text. *ACM Comput. Surv.* **51**, 85 (2018).

[28] Phadke, S. & Mitra, T. Many faced hate: a cross platform study of content framing and information sharing by online hate groups. In *Proceedings of the 2020 CHI Conference on Human Factors in Computing Systems* 1–13 (Association for Computing Machinery, 2020).

[29] Xia, Y., Zhu, H., Lu, T., Zhang, P. & Gu, N. Exploring antecedents and consequences of toxicity in online discussions: a case study on Reddit. *Proc. ACM Hum. Comput. Interact.* **4**, 108 (2020).

[30] Sipka, A., Hannak, A. & Urman, A. Comparing the language of qanon-related content on Parler, GAB, and Twitter. In *Proc. 14th ACM Web Science Conference 2022* 411–421 (Association for Computing Machinery, 2022).

[31] Fortuna, P., Soler, J. & Wanner, L. Toxic, hateful, offensive or abusive? What are we really classifying? An empirical analysis of hate speech datasets. In *Proc. 12th Language Resources and Evaluation Conference* (eds Calzolari, E. et al.) 6786–6794 (European Language Resources Association, 2020).

[32] Davidson, T., Warmsley, D., Macy, M. & Weber, I. Automated hate speech detection and the problem of offensive language. In *Proc. International AAAI Conference on Web and Social Media* 11 (Association for the Advancement of Artificial Intelligence, 2017).

[33] Kolhatkar, V. et al. The SFU opinion and comments corpus: a corpus for the analysis of online news comments. *Corpus Pragmat.* **4**, 155–190 (2020). 32685909 10.1007/s41701-019-00065-w PMC7357677

[34] Lees, A. et al. A new generation of perspective API: efficient multilingual character-level transformers. In *KDD'22: The 28th ACM SIGKDD Conference on Knowledge Discovery and Data Mining* 3197–3207 (Association for Computing Machinery, 2022).

[35] Vidgen, B. & Derczynski, L. Directions in abusive language training data, a systematic review: garbage in, garbage out. *PLoS ONE* **15**, e0243300 (2020). 33370298 10.1371/journal.pone.0243300 PMC7769249

[36] Ross, G. J. & Jones, T. Understanding the heavy-tailed dynamics in human behavior. *Phys. Rev. E* **91**, 062809 (2015). 10.1103/PhysRevE.91.062809 26172756

[37] Choi, D., Chun, S., Oh, H., Han, J. & Kwon, T. T. Rumor propagation is amplified by echo chambers in social media. *Sci. Rep.* **10**, 310 (2020). 31941980 10.1038/s41598-019-57272-3 PMC6962360

[38] Beel, J., Xiang, T., Soni, S. & Yang, D. Linguistic characterization of divisive topics online: case studies on contentiousness in abortion, climate change, and gun control. *In Proc. International AAAI Conference on Web and Social Media* Vol. 16, 32–42 (Association for the Advancement of Artificial Intelligence, 2022).

[39] Saveski, M., Roy, B. & Roy, D. The structure of toxic conversations on Twitter. In *Proc. Web Conference 2021* (eds Leskovec, J. et al.) 1086–1097 (Association for Computing Machinery, 2021).

[40] Juul, J. L. & Ugander, J. Comparing information diffusion mechanisms by matching on cascade size. *Proc. Natl Acad. Sci. USA* **118**, e2100786118 (2021). 34750252 10.1073/pnas.2100786118 PMC8609637

[41] Fariello, G., Jemielniak, D. & Sulkowski, A. Does Godwin’s law (rule of Nazi analogies) apply in observable reality? An empirical study of selected words in 199 million Reddit posts. *N. Media Soc.* **26**, 14614448211062070 (2021).

[42] Qiu, J., Lin, Z. & Shuai, Q. Investigating the opinions distribution in the controversy on social media. *Inf. Sci.* **489**, 274–288 (2019).

[43] Garimella, K., Morales, G. D. F., Gionis, A. & Mathioudakis, M. Quantifying controversy on social media. *ACM Trans. Soc. Comput.* **1**, 3 (2018).

[44] NLPTown. bert-base-multilingual-uncased-sentiment, huggingface.co/nlptown/bert-base-multilingual-uncased-sentiment (2023).

[45] Ta, H. T., Rahman, A. B. S., Najjar, L. & Gelbukh, A. *Transfer Learning from Multilingual DeBERTa for Sexism Identification* CEUR Workshop Proceedings Vol. 3202 (CEUR-WS, 2022).

[46] Kleinberg, J. Bursty and hierarchical structure in streams. *Data Min. Knowl. Discov.* **7**, 373–397 (2003).

[47] Zollo, F. et al. Debunking in a world of tribes. *PLoS ONE* **12**, e0181821 (2017). 28742163 10.1371/journal.pone.0181821 PMC5524392

[48] Albrecht, D. Vaccination, politics and COVID-19 impacts. *BMC Publ. Health* **22**, 96 (2022). 10.1186/s12889-021-12432-x PMC8758893 35031053

[49] Falkenberg, M. et al. Growing polarization around climate change on social media. *Nat. Clim. Change* **12**, 1114–1121 (2022).

[50] Schmidt, A. L., Zollo, F., Scala, A., Betsch, C. & Quattrociocchi, W. Polarization of the vaccination debate on Facebook. *Vaccine* **36**, 3606–3612 (2018). 29773322 10.1016/j.vaccine.2018.05.040

[51] Schmidt, A. L. et al. Anatomy of news consumption on Facebook. *Proc. Natl Acad. Sci. USA* **114**, 3035–3039 (2017). 28265082 10.1073/pnas.1617052114 PMC5373354

[52] Del Vicario, M., Zollo, F., Caldarelli, G., Scala, A. & Quattrociocchi, W. Mapping social dynamics on Facebook: the brexit debate. *Soc. Netw.* **50**, 6–16 (2017).

[53] Hunnicutt, T. & Dave, P. Gab.com goes offline after Pittsburgh synagogue shooting. *Reuters*, www.reuters.com/article/uk-pennsylvania-shooting-gab-idUKKCN1N20QN (29 October 2018).

[54] Valensise, C. M. et al. Lack of evidence for correlation between COVID-19 infodemic and vaccine acceptance. Preprint at arxiv.org/abs/2107.07946 (2021).

[55] Quattrociocchi, A., Etta, G., Avalle, M., Cinelli, M. & Quattrociocchi, W. in *Social Informatics* (eds Hopfgartner, F. et al.) 245–256 (Springer, 2022).

[56] Mekacher, A. & Papasavva, A. “I can’t keep it up” a dataset from the defunct voat.co news aggregator. In *Proc. International AAAI Conference on Web and Social Media* Vol. 16, 1302–1311 (AAAI, 2022).

[57] *Facebook Community Standards*, transparency.fb.com/policies/community-standards/hate-speech/ (Facebook, 2023).

[58] Rosen, G. & Lyons, T. Remove, reduce, inform: new steps to manage problematic content. *Meta*, about.fb.com/news/2019/04/remove-reduce-inform-new-steps/ (10 April 2019).

[59] *Vulgar Language Policy*, support.google.com/youtube/answer/10072685? (YouTube, 2023).

[60] *Harassment & Cyberbullying Policies*, support.google.com/youtube/answer/2802268 (YouTube, 2023).

[61] *Hate Speech Policy*, support.google.com/youtube/answer/2801939 (YouTube, 2023).

[62] *How Does YouTube Enforce Its Community Guidelines?*, www.youtube.com/intl/enus/howyoutubeworks/policies/community-guidelines/enforcing-community-guidelines (YouTube, 2023).

[63] *The Twitter Rules*, help.twitter.com/en/rules-and-policies/twitter-rules (Twitter, 2023).

[64] *Hateful Conduct*, help.twitter.com/en/rules-and-policies/hateful-conduct-policy (Twitter, 2023).

[65] Gorwa, R., Binns, R. & Katzenbach, C. Algorithmic content moderation: technical and political challenges in the automation of platform governance. *Big Data Soc.* **7**, 2053951719897945 (2020).

[66] *Our Range of Enforcement Options*, help.twitter.com/en/rules-and-policies/enforcement-options (Twitter, 2023).

[67] Elliott, V. & Stokel-Walker, C. Twitter’s moderation system is in tatters. *WIRED* (17 November 2022).

[68] *Reddit Content Policy*, www.redditinc.com/policies/content-policy (Reddit, 2023).

[69] *Promoting Hate Based on Identity or Vulnerability*, www.reddithelp.com/hc/en-us/articles/360045715951 (Reddit, 2023).

[70] Malik, A. Reddit acqui-hires team from ML content moderation startup Oterlu. *TechCrunch*, tcrn.ch/3yeS2Kd (4 October 2022).

[71] *Terms of Service*, telegram.org/tos (Telegram, 2023).

[72] Durov, P. The rules of @telegram prohibit calls for violence and hate speech. We rely on our users to report public content that violates this rule. *Twitter*, twitter.com/durov/status/917076707055751168?lang=en (8 October 2017).

[73] *Telegram Privacy Policy*, telegram.org/privacy (Telegram, 2023).

[74] *Terms of Service*, gab.com/about/tos (Gab, 2023).

[75] Salzenberg, C. & Spafford, G. *What is Usenet?*, www0.mi.infn.it/∼calcolo/Wis usenet.html (1995).

[76] Castelle, M. The linguistic ideologies of deep abusive language classification. In *Proc. 2nd Workshop on Abusive Language Online (ALW2)* (eds Fišer, D. et al.) 160–170, aclanthology.org/W18-5120 (Association for Computational Linguistics, 2018).

[77] Tontodimamma, A., Nissi, E. & Sarra, A. E. A. Thirty years of research into hate speech: topics of interest and their evolution. *Scientometrics* **126**, 157–179 (2021).

[78] Sap, M. et al. Annotators with attitudes: how annotator beliefs and identities bias toxic language detection. In *Proc. 2022 Conference of the North American Chapter of the Association for Computational Linguistics: Human Language Technologies* (eds. Carpuat, M. et al.) 5884–5906 (Association for Computational Linguistics, 2022).

[79] Pavlopoulos, J., Sorensen, J., Dixon, L., Thain, N. & Androutsopoulos, I. Toxicity detection: does context really matter? In *Proc. 58th Annual Meeting of the Association for Computational Linguistics* (eds Jurafsky, D. et al.) 4296–4305 (Association for Computational Linguistics, 2020).

[80] Yin, W. & Zubiaga, A. Hidden behind the obvious: misleading keywords and implicitly abusive language on social media. *Online Soc. Netw. Media* **30**, 100210 (2022).

[81] Sap, M., Card, D., Gabriel, S., Choi, Y. & Smith, N. A. The risk of racial bias in hate speech detection. In *Proc. 57th Annual Meeting of the Association for Computational Linguistics* (eds Kohonen, A. et al.) 1668–1678 (Association for Computational Linguistics, 2019).

[82] Rosenblatt, L., Piedras, L. & Wilkins, J. Critical perspectives: a benchmark revealing pitfalls in PerspectiveAPI. In *Proc. Second Workshop on NLP for Positive Impact (NLP4PI)* (eds Biester, L. et al.) 15–24 (Association for Computational Linguistics, 2022).

[83] DiMaggio, P., Evans, J. & Bryson, B. Have American’s social attitudes become more polarized? *Am. J. Sociol.* **102**, 690–755 (1996).

[84] Fiorina, M. P. & Abrams, S. J. Political polarization in the American public. *Annu. Rev. Polit. Sci.* **11**, 563–588 (2008).

[85] Iyengar, S., Gaurav, S. & Lelkes, Y. Affect, not ideology: a social identity perspective on polarization. *Publ. Opin. Q.* **76**, 405–431 (2012).

[86] Cota, W., Ferreira, S. & Pastor-Satorras, R. E. A. Quantifying echo chamber effects in information spreading over political communication networks. *EPJ Data Sci.* **8**, 38 (2019).

[87] Bessi, A. et al. Users polarization on Facebook and Youtube. *PLoS ONE* **11**, e0159641 (2016). 27551783 10.1371/journal.pone.0159641 PMC4994967

[88] Bessi, A. et al. Science vs conspiracy: collective narratives in the age of misinformation. *PLoS ONE* **10**, e0118093 (2015). 25706981 10.1371/journal.pone.0118093 PMC4338055

[89] Himelboim, I., McCreery, S. & Smith, M. Birds of a feather tweet together: integrating network and content analyses to examine cross-ideology exposure on Twitter. *J. Comput. Med. Commun.* **18**, 40–60 (2013).

[90] An, J., Quercia, D. & Crowcroft, J. Partisan sharing: Facebook evidence and societal consequences. In *Proc. Second ACM Conference on Online Social Networks, COSN*′*14* 13–24 (Association for Computing Machinery, 2014).

[91] Mann, H. B. & Whitney, D. R. On a test of whether one of two random variables is stochastically larger than the other. *Ann. Math. Stat.* **18**, 50–60 (1947).

