# Supplementary material for: Persistent interaction patterns across social media platforms and over time
Source: Nature. 2024 Mar 20;628(8008):582–9. doi: 10.1038/s41586-024-07229-y (PMC11023927; doi:10.1038/s41586-024-07229-y)
Supplement: Supplementary file 1 — Supplementary Information 1–4, including details regarding data collection for validation dataset, Supplementary Figs. 1–3, Supplementary Tables 1–17 and software and coding specifications. [file 41586_2024_7229_MOESM1_ESM.pdf]

nature portfolio

https://doi.org/10.1038/s41586-024-07229-y

Supplementary information

Persistent interaction patterns across social media platforms and over time

In the format provided by the authors and unedited

Persistent Interaction Patterns Across Social Media Platforms and Over Time

Michele Avalle[1†], Niccolò Di Marco[1†], Gabriele Etta[1†], Emanuele Sangiorgio[2], Shayan Alipour[1], Anita Bonetti[1], Lorenzo Alvisi[1], Antonio Scala[3], Andrea Baronchelli[4,5], Matteo Cinelli[1*] and Walter Quattrociocchi[1*]

[1*]Department of Computer Science, Sapienza University of Rome, Viale Regina Elena 295, Rome, 00161, Italy.
[2]Department of Social Sciences and Economics, Sapienza University of Rome, Piazzale Aldo Moro 5, Rome, 00185, Italy.
[3]Institute of Complex Systems, CNR, Via dei Taurini 19, Rome, 00185, Italy.
[4]City University of London, Department of Mathematics, London, EC1V0HB, UK.
[5]The Alan Turing Institute 96 Euston Road, London, NW12DB, UK.

*Corresponding author(s). E-mail(s): matteo.cinelli@uniroma1.it; walter.quattrociocchi@uniroma1.it;
†These authors contributed equally to this work.

Contents

1 Data Collection for validation dataset **2**

2 Supplementary Tables **3**

3 Supplementary Figures **16**

4 Software and Coding specifications **19**
4.1 Softwares Employed 20
4.2 List of R packages 20

1 Data Collection for validation dataset

Facebook We collect data regarding Sports and Film topics from datasets of Italian news outlet page posts and comments described in [1]. Concerning the Sports topic, we collect a total of $\sim 494K$ comments ranging from 2015-01-01 to 2017-08-19 and using the following keywords: {*sport, serie A, serieA, calcio, tennis, pallavolo, nuoto*}. For the Film topic instead, we collect a total of $\sim 493K$ comments ranging from 2015-01-01 to 2017-08-10, using the following keywords: {*film, cinema, regista, registi, cinematografia*}.

Reddit We collect data from subreddits which are known for hosting conversations about a multitude of topics, namely *r/AskReddit* and *r/IAmA*, together with a third subreddit, named *r/Movies*, which contains conversations about movies. Concerning *r/AskReddit*, we collect a total of ($\sim 2.3M$ comments) from 2021-02-01 to2021-02-15. For *r/IAmA*, instead, we collect a total of $\sim 500K$ comments between 2021-02-01 and 2022-12-31). Finally, for *r/movies*, we collect $\sim 11M$ comments from 2021-02-01 to 2022-12-31.

Telegram We collect data related to *Crypto* topic from a channel named *WatcherGuru* from 2021-08-13 to 2023/06/22, for a total of $\sim 150K$ comments.

Twitter We collect data about the *Game of Thrones* (GoT) and *NASA* topics. Concerning the first, we retrieve all comments containing the hashtag #gameofthrones from 2019-01-01 to 2022-12-24, accounting for $\sim 442K$ comments. For NASA, instead, we collect comments from posts shared by the official NASA account on Twitter between 2019-01-01 and 2022-12-29 for a total of $\sim 346K$ comments.

Voat We collect data from two Q&A generic subverses, namely *askvoat* and *whatever*. Concerning the first, we collect $\sim 612K$ comments from 2014-06-19 to 2020-12-25, whilst for the second, we collect $\sim 1.4M$ comments from 2015-05-31 to 2020-12-25.

YouTube Data collection on YouTube is performed using the YouTube Data API. We collect videos that contain at least one search term from the following list of keywords related to football: {*SerieA, SerieATim, VAR, Napoli, ForzaNapoliSempre, RangersNapoli, Atalanta, GoAtalantaGo, ForzaAtalanta, Milan, ACMilan, SempreMilan, Udinese, ForzaUdinese, AlèUdin, Inter, IMInter, ForzaInter, Forza Lazio, La Lazio, SSLazio, CMonEagles, ASRoma, Juventus, JuventusFC, Juve, ForzaJuve, IlTorino, Forza Torino, SFT, Salernitana, forzagranata, Fiorentina, forzaviola, ACFFiorentina, IlBologna, ForzaBologna, ForzaBFC, WeAreOne, IlSassuolo, Forza Sassuolo, ForzaSasol,*

LEmpoli, ForzaEmpoli, EmpoliFC, EmpoliFootballChannel, HellasVerona, Hellas, DaiVerona, HVFC, LoSpezia, ForzaSpezia, SpeziaCalcio, IlLecce, ForzaLecce, avantilecce, Cremonese, SolAmAi, ForzaGrigiorossi, DaiCremo, Sampdoria, FORZADORIA, Il Monza, Forza Monza, ACMonza, Monza, InsiemealMonza}. Then, we retrieved all the comments for each video, resulting in a total of $\sim 1M$ comments between 2022-08-02 and 2023-04-07. Consistently with the previously mentioned keywords search, we collect another dataset using only the keyword {*carbonara*} during a period ranging between 2018/01/05 and 2023/07/02, for a total of $\sim 700K$ comments.

2 Supplementary Tables

Table S1: **User activity distributions show heavy-tailed behaviour.** The table contains the mean μ, standard deviation σ, skewness γ, kurtosis β, minimum m, maximum M and mean over maximum ratio values of the distributions shown in Fig.1. The very high values of γ and β suggest that a right heavy-tail is present in all the distributions. Moreover, the very low values of $\frac{\mu}{M}$ provide further evidence for the long tail exhibited by all of them.

Dataset	μ	σ	γ	β	m	M	$\frac{\mu}{M} \cdot 10^3$
Facebook Brexit	1.843	3.314	14.912	432.213	1	199	9.262
Facebook News	5.967	46.509	175.370	85363.523	1	40563	0.147
Facebook Vaccines	5.335	50.687	216.401	74222.282	1	20469	0.261
Gab Feed	87.761	711.604	25.168	1036.119	1	59397	1.478
Reddit Climate Change	2.664	7.281	40.011	3119.201	1	684	3.895
Reddit Conspiracy	8.388	98.832	255.494	72719.500	1	28318	0.296
Reddit News	3.546	9.883	16.811	539.386	1	647	5.481
Reddit Science	2.598	29.224	396.043	171147.901	1	12745	0.204
Reddit Vaccines	12.800	71.166	17.648	411.305	1	2318	5.522
Telegram Conspiracy	12.203	62.824	51.373	5462.725	1	9568	1.275
Telegram News	49.630	320.969	30.318	1394.893	1	18517	2.680
Telegram Politics	99.935	605.414	23.158	736.514	1	22651	4.412
Twitter Climate Change	2.714	7.700	77.792	15857.718	1	2616	1.037
Twitter News	5.548	39.023	539.434	490973.585	1	37263	0.149
Twitter Vaccines	4.169	16.133	138.508	53969.078	1	10246	0.407
Usenet Conspiracy	5.548	50.099	80.142	9523.899	1	6662	0.833
Usenet News	7.752	86.858	160.344	32776.435	1	18107	0.428
Usenet Politics	11.221	88.391	71.253	8959.542	1	15632	0.718
Usenet Talk	11.573	97.128	50.215	4570.797	1	13724	0.843
Voat Conspiracy	37.076	301.851	37.342	2342.212	1	25706	1.442
Voat News	15.808	172.533	79.570	10437.417	1	28664	0.551
Voat Politics	16.318	172.781	71.644	7852.781	1	23632	0.691
YouTube Climate Change	1.940	4.247	30.906	2314.805	1	625	3.104
YouTube News	4.764	36.292	296.198	255275.719	1	36900	0.129
YouTube Vaccines	2.936	13.012	102.905	25721.972	1	4797	0.612

Table S2: **Thread length distributions show heavy-tailed behaviour.** The table contains the mean μ, standard deviation σ, skewness γ, kurtosis β, minimum m, maximum M and mean over maximum ratio values of the distributions shown in Fig.1a in ED. The very high values of γ and β suggest that a right heavy-tail is present in all the distributions. Moreover, the very low values of $\frac{\mu}{M}$ provide further evidence for their long tail.

Dataset	μ	σ	γ	β	m	M	$\frac{\mu}{M} \cdot 10^3$
Facebook Brexit	109.588	362.950	12.757	247.364	1	8986	12.195
Facebook News	52.581	360.100	95.318	20135.944	1	136406	0.385
Facebook Vaccines	13.485	36.459	34.907	2827.570	1	4003	3.369
Gab Feed	3.889	11.144	30.630	2550.265	1	2183	1.782
Reddit Climate Change	13.970	47.460	6.011	44.221	1	481	29.044
Reddit Conspiracy	22.153	48.062	5.449	38.573	1	481	46.056
Reddit News	49.959	98.397	2.487	8.132	1	445	112.268
Reddit Science	19.398	57.378	4.473	24.275	1	491	39.507
Reddit Vaccines	14.641	22.131	4.474	35.734	1	341	42.936
Telegram Conspiracy	56.257	103.770	23.173	1167.402	1	7580	7.422
Telegram News	29.328	55.120	5.473	54.222	1	1383	21.206
Telegram Politics	22.084	41.439	10.556	225.954	1	1534	14.396
Twitter Climate Change	74.613	1068.886	119.338	22036.559	1	232837	0.320
Twitter News	97.013	493.960	52.815	5130.872	1	69417	1.398
Twitter Vaccines	393.399	2638.999	44.737	3567.687	1	329653	1.193
Usenet Conspiracy	4.393	12.474	28.288	1848.763	1	1132	3.881
Usenet News	5.829	17.918	22.216	959.125	1	1244	4.685
Usenet Politics	6.056	37.024	299.079	129004.274	1	16309	0.371
Usenet Talk	5.593	17.698	17.901	592.392	1	1095	5.108
Voat Conspiracy	10.253	14.142	4.949	70.838	1	636	16.121
Voat News	8.185	13.985	5.329	55.630	1	386	21.204
Voat Politics	7.574	13.208	5.399	53.931	1	358	21.158
Youtube Climate Change	93.804	737.191	37.626	1791.166	1	38793	2.418
Youtube News	190.361	945.639	82.539	13161.303	1	178889	1.064
Youtube Vaccines	187.242	610.426	18.179	755.517	1	34106	5.490

Table S3: **User lifetime distributions show heavy-tailed behaviour.** The table contains the mean μ, standard deviation σ, skewness γ, kurtosis β, minimum m, maximum M and mean over maximum ratio values of the distributions shown in Fig.1b in ED. The very high values of γ and β suggest that a right heavy-tail is present in all the distributions. Moreover, the very low values of $\frac{\mu}{M}$ provide further evidence for their long tail.

Dataset	μ	σ	γ	β	m	M	$\frac{\mu}{M} \cdot 10^3$
Facebook Brexit	8.458	27.446	3.974	19.077	0	205	41.259
Facebook News	150.865	312.934	2.865	12.296	0	2379	63.415
Facebook Vaccines	187.382	396.198	2.797	11.636	0	2706	69.247
Gab Feed	94.241	174.820	2.171	6.929	0	805	117.069
Reddit Climate Change	55.493	238.965	5.260	30.318	0	1804	30.761
Reddit Conspiracy	109.709	277.915	3.487	14.948	0	1671	65.655
Reddit News	44.965	93.334	2.220	6.762	0	364	123.530
Reddit Science	140.262	326.552	2.538	8.476	0	1802	77.837
Reddit Vaccines	44.802	126.924	4.490	29.140	0	1583	28.302
Telegram Conspiracy	92.997	165.000	2.131	6.960	0	1204	77.240
Telegram News	111.731	174.069	2.151	9.394	0	1688	66.191
Telegram Politics	233.781	281.810	1.479	6.410	0	1962	119.154
Twitter Climate Change	19.747	75.233	5.500	33.594	0	996	19.827
Twitter News	110.800	197.114	1.877	5.544	0	1054	105.123
Twitter Vaccines	50.042	120.911	6.688	84.010	0	3437	14.560
Usenet Conspiracy	39.449	157.473	7.797	88.666	0	3532	11.169
Usenet News	53.808	186.146	6.714	69.470	0	4137	13.006
Usenet Politics	65.119	210.660	6.048	54.987	0	4704	13.843
Usenet Talk	69.355	227.014	6.110	55.339	0	4270	16.242
Voat Conspiracy	113.371	231.480	2.143	6.501	0	1081	104.876
Voat News	87.741	287.198	4.306	22.930	0	2373	36.975
Voat Politics	98.507	297.648	3.938	19.564	0	2371	41.547
Youtube Climate Change	33.279	175.596	7.720	73.824	0	2603	12.785
Youtube News	224.278	535.427	3.640	19.731	0	5595	40.085
Youtube Vaccines	28.551	69.920	3.127	13.809	0	603	47.348

Table S4: **Thread lifetime distributions show heavy-tailed behaviour.** The table contains the mean μ, standard deviation σ, skewness γ, kurtosis β, minimum m, maximum M and mean over maximum ratio values of the distributions shown in Fig.1c in ED. The very high values of γ and β suggest that a right heavy-tail is present in all the distributions. Moreover, the very low values of $\frac{\mu}{M}$ provide further evidence for their long tail.

Dataset	μ	σ	γ	β	m	M	$\frac{\mu}{M} \cdot 10^3$
Facebook Brexit	4.028	13.898	6.216	47.714	0	167	24.119
Facebook News	10.018	75.216	12.640	209.703	0	2400	4.174
Facebook Vaccines	12.656	94.661	12.575	196.556	0	2481	5.101
Gab Feed	1.329	15.381	22.043	627.516	0	791	1.680
Reddit Climate Change	1.373	19.584	57.393	3746.499	0	1293	1.062
Reddit Conspiracy	6.863	79.606	17.290	313.775	0	1744	3.935
Reddit News	1.379	6.775	12.223	190.254	0	149	9.254
Reddit Science	1.564	9.932	11.333	151.991	0	179	8.736
Reddit Vaccines	7.744	44.243	13.267	225.489	0	991	7.814
Telegram Conspiracy	14.553	45.247	7.566	76.725	0	737	19.746
Telegram News	2.471	38.157	23.953	692.321	0	1417	1.744
Telegram Politics	2.351	21.116	21.942	675.352	0	881	2.669
Twitter Climate Change	10.610	65.619	8.703	87.204	0	1030	10.301
Twitter News	7.501	47.665	11.643	159.696	0	1034	7.254
Twitter Vaccines	53.967	165.962	4.629	39.334	0	4382	12.316
Usenet Conspiracy	4.698	28.200	41.912	3388.995	0	2831	1.660
Usenet News	2.978	21.940	73.221	8983.108	0	3419	0.871
Usenet Politics	3.289	21.696	51.650	5004.203	0	3283	1.002
Usenet Talk	3.546	23.748	59.778	6438.581	0	3924	0.904
Voat Conspiracy	0.877	6.815	44.525	5847.673	0	1057	0.829
Voat News	0.491	7.031	171.089	48056.295	0	2110	0.233
Voat Politics	0.440	6.978	174.368	47319.457	0	1998	0.220
Youtube Climate Change	121.841	350.261	4.040	20.166	0	2659	45.822
Youtube News	508.682	807.791	2.004	6.983	0	5820	87.402
Youtube Vaccines	50.954	74.673	2.361	10.631	0	607	83.944

Table S5: The majority of users wrote at least one toxic comment. For each dataset, the table contains the fraction f of users (having at least 11 comments) who posted at least one toxic comment.

Dataset	f
YouTube Climate Change	0.48
YouTube News	0.71
YouTube Vaccines	0.43
Twitter Climate Change	0.51
Twitter News	0.62
Twitter Vaccines	0.59
Telegram Conspiracy	0.77
Telegram News	0.47
Telegram Politics	0.70
Reddit Climate Change	0.51
Reddit Conspiracy	0.70
Reddit News	0.71
Reddit Science	0.20
Reddit Vaccines	0.53
Facebook Brexit	0.59
Facebook Vaccines	0.53
Facebook News	0.58
Gab Feed	0.80
Usenet Conspiracy	0.53
Usenet News	0.63
Usenet Politics	0.66
Usenet Talk	0.51
Voat Conspiracy	0.85
Voat News	0.90
Voat Politics	0.91

Table S6: **Validation dataset breakdown**. TP, TD and TI indicate, respectively, the percentage of toxic comments in a dataset labelled by Perspective, Detoxify and IMSYPP.

Dataset	Time range	Comments	Threads	Users	TP	TD	TI
Facebook Film	2015-01-01 - 2017-08-10	493825	13385	305415	0.03	0.09	0.13
Facebook Sports	2015-01-01 - 2017-08-19	494275	19632	271575	0.04	0.11	0.18
Reddit AskReddit	2021-02-01 - 2021-02-15	2380486	156168	492891	0.07	0.11	0.16
Reddit IAmA	2021-02-01 - 2022-12-31	501507	13027	194197	0.03	0.06	0.12
Reddit Movies	2021-02-01 - 2023-01-01	11597305	159976	1263209	0.05	0.10	0.18
Telegram Crypto	2021-08-13 - 2023-06-22	147610	4551	19372	0.05	0.09	0.20
Twitter Got	2019-01-01 - 2022-12-24	442709	62488	222514	0.02	0.04	0.05
Twitter NASA	2019-01-01 - 2022-12-29	346157	22906	187061	0.02	0.03	0.06
Voat Askvoat	2014-06-19 - 2020-12-25	612668	50389	55242	0.12	0.19	0.28
Voat Whatever	2015-05-31 - 2020-12-25	1366671	177278	76905	0.20	0.27	0.40
Youtube Carbonara	2018-01-05 - 2023-07-02	699670	3615	545174	0.03	0.04	0.04
Youtube Football	2022-08-02 - 2023-04-07	948918	16887	165706	0.02	0.08	0.13

Table S7: **Dataset subsets for the analysis of participation and toxicity along threads.** For each dataset, it is reported the number of conversations, along with their minimum and maximum size, which correspond to the 0.7 and 1 values of the normalized log-binning.

Dataset	Threads	Min. size	Max. size
Facebook Brexit	711	140	8986
Facebook News	25488	1424	136406
Facebook Vaccines	3959	81	4003
Gab Feed	5931	104	2183
Reddit Climate Change	325	51	481
Reddit Conspiracy	2499	62	481
Reddit News	1529	58	445
Reddit Science	2058	63	491
Reddit Vaccines	730	26	341
Telegram Conspiracy	3814	115	7580
Telegram News	2723	77	1383
Telegram Politics	1817	62	1534
Twitter Climate Change	2076	836	232837
Twitter News	3382	474	69417
Twitter Vaccines	5681	1904	329653
Usenet Conspiracy	798	31	1132
Usenet News	1165	50	1244
Usenet Politics	1824	87	16309
Usenet Talk	2472	74	1095
Voat Conspiracy	5619	34	636
Voat News	6064	40	386
Voat Politics	5134	37	358
YouTube Climate Change	800	182	38793
YouTube News	6244	732	178889
YouTube Vaccines	1717	321	34106

Table S8: **There is no difference in how participation evolves in toxic and non-toxic conversations.** For each dataset, we report the $p-$values associated with the interaction effect between toxic and non-toxic threads in the participation regression.

Dataset	p
Facebook Brexit	0.553
Facebook News	0.107
Facebook Vaccines	< 0.001
Gab Feed	0.874
Reddit Climate Change	0.062
Reddit Conspiracy	0.243
Reddit News	0.279
Reddit Science	0.434
Reddit Vaccines	0.666
Telegram Conspiracy	0.005
Telegram News	0.976
Telegram Politics	0.847
Twitter Climate Change	0.069
Twitter News	0.034
Twitter Vaccines	0.026
Usenet Conspiracy	0.653
Usenet News	0.391
Usenet Politics	0.683
Usenet Talk	0.873
Voat Conspiracy	0.989
Voat News	0.716
Voat Politics	0.700
YouTube Climate Change	0.939
YouTube News	0.347
YouTube Vaccines	0.113

Table S9: **Perspective is a valid classificator also for Usenet.** For each fraction of non-toxic comments (column headers), we report the fraction of slopes that lie outside the interval $(-|s|, |s|)$, where s is the slope of the observed trend. The trend is considered significantly different if the associated fraction is less than 0.05.

Dataset	1%	3%	5%	7%	9%	11%	13%	15%
Usenet Politics	1.00	1.00	1.00	1.00	1.00	1.00	1.00	1.00
Usenet Conspiracy	0.98	0.82	0.74	0.67	0.61	0.54	0.49	0.42
Usenet Talk	< 0.01	< 0.01	< 0.01	< 0.01	< 0.01	< 0.01	< 0.01	< 0.01
Usenet News	1.00	1.00	1.00	1.00	1.00	1.00	1.00	1.00

Table S10: **Validation dataset subsets for the analysis of participation and toxicity along threads.** For each dataset, it is reported the number of conversations, along with their minimum and maximum size, which correspond to the 0.7 and 1 values of the normalized log-binning.

Dataset	Threads	Min. size	Max. size
Facebook Film	919	114	7894
Facebook Sports	1083	91	10689
Reddit Askreddit	365	395	33506
Reddit Iama	562	175	12275
Reddit Movies	1937	920	90977
Telegram Crypto	1255	40	574
Twitter Got	567	81	20264
Twitter Nasa	611	89	6481
Voat Askvoat	3072	37	429
Voat Whatever	6233	37	387
Youtube Carbonara	694	199	20249
Youtube Football	2283	115	2024

Table S11: **No difference in participation trends in toxic and non-toxic conversations for the validation dataset.** For each validation dataset and classifier, we report the *p*-value associated with the interaction effect between toxic and non-toxic threads in the participation regression.

Dataset	Perspective	Detoxify	Imsypp
Facebook Film	0.705	0.437	0.461
Facebook Sports	0.870	0.731	0.456
Reddit Askreddit	0.218	0.679	0.283
Reddit Iama	0.987	0.543	0.475
Reddit Movies	0.248	0.161	0.167
Telegram Crypto	0.493	0.627	0.890
Twitter Got	0.052	0.673	0.736
Twitter Nasa	0.002	0.004	0.001
Voat Askvoat	0.193	0.298	0.086
Voat Whatever	0.288	0.300	0.196
YouTube Carbonara	0.648	0.621	0.360
YouTube Football	0.100	0.254	0.564

Table S12: **Toxicity does not tend to increase with user engagement.** Burst analysis of activity in conversations (see ED tab. 4) for the validation dataset, using the three toxicity detectors.

Dataset	N. of Threads	Peak >Pre	Peak >Post	Post >Pre	Detector
Reddit Askreddit	17879	0.000	0.000	0.000	Perspective
Reddit Iama	1403	0.000	1.000	0.000	Perspective
Reddit Movies	5000	0.000	0.000	0.000	Perspective
Voat Askvoat	8597	0.000	0.000	0.002	Perspective
Voat Whatever	16537	0.000	0.000	0.000	Perspective
Telegram Crypto	2167	0.000	0.043	0.000	Perspective
Telegram Got	2912	0.000	0.032	0.000	Perspective
Telegram Nasa	2580	0.000	1.000	0.000	Perspective
Facebook Film	3990	0.000	0.860	0.000	Perspective
Facebook Sports	4588	0.000	0.147	0.000	Perspective
YouTube Carbonara	1864	0.000	1.000	0.000	Perspective
YouTube Football	6824	0.000	0.002	0.000	Perspective
Reddit Askreddit	17879	0.000	0.000	0.000	Detoxify
Reddit Iama	1403	0.000	1.000	0.000	Detoxify
Reddit Movies	5000	0.000	0.000	0.000	Detoxify
Voat Askvoat	8597	0.000	0.005	0.049	Detoxify
Voat Whatever	16537	0.000	0.022	0.000	Detoxify
Telegram Crypto	2167	0.000	0.004	0.000	Detoxify
Twitter Got	2912	0.000	0.117	0.000	Detoxify
Twitter Nasa	2580	0.000	1.000	0.000	Detoxify
Facebook Film	3990	0.000	0.896	0.000	Detoxify
Facebook Sports	4588	0.000	0.267	0.000	Detoxify
YouTube Carbonara	1864	0.000	0.713	0.000	Detoxify
YouTube Football	6824	0.000	0.000	0.000	Detoxify
Reddit Askreddit	17879	0.000	0.012	0.000	IMSYPP
Reddit Iama	1403	0.000	1.000	0.000	IMSYPP
Reddit Movies	5000	0.025	0.471	0.590	IMSYPP
Voat Askvoat	8597	0.004	0.488	0.110	IMSYPP
Voat Whatever	16537	0.002	0.764	0.019	IMSYPP
Telegram Crypto	2167	0.000	0.003	0.000	IMSYPP
Twitter Got	2912	0.000	0.331	0.000	IMSYPP
Twitter Nasa	2580	0.000	0.772	0.000	IMSYPP
Facebook Film	3990	0.000	1.000	0.000	IMSYPP
Facebook Sports	4588	0.000	1.000	0.000	IMSYPP
YouTube Carbonara	1864	0.000	1.000	0.000	IMSYPP
YouTube Football	6824	0.000	0.020	0.000	IMSYPP

Table S13: **Validation dataset: toxicity increases with conversation length.** Results of Mann-Kendall test on the toxicity trends T_o and slope from linear regression β_0 of the validation dataset for Perspective Detoxify and IMSYPP.

Dataset	Perspective			Detoxify			IMSYPP		
	T_o	β_0 $\cdot 10^{-3}$	p	T_o	β_0 $\cdot 10^{-3}$	p	T_o	β_0 $\cdot 10^{-3}$	p
Facebook Film	$\uparrow$	26.514	< 0.001	$\uparrow$	43.801	< 0.001	$\uparrow$	76.734	< 0.001
Facebook Sports	$\uparrow$	43.609	< 0.001	$\uparrow$	79.326	< 0.001	$\uparrow$	145.053	< 0.001
Reddit Askreddit	$\uparrow$	19.875	0.021	$\uparrow$	40.909	0.003	$\uparrow$	71.521	< 0.001
Reddit Iama	$\uparrow$	22.880	< 0.001	$\uparrow$	50.688	< 0.001	$\uparrow$	111.957	< 0.001
Reddit Movies	$\uparrow$	20.437	0.027	$\uparrow$	43.326	< 0.001	$\uparrow$	81.102	< 0.001
Telegram Crypto	$\uparrow$	29.474	< 0.001	$\uparrow$	33.382	< 0.001	$\uparrow$	107.940	< 0.001
Twitter Got	$\uparrow$	15.237	0.010	$\uparrow$	25.348	< 0.001	$\uparrow$	43.010	< 0.001
Twitter Nasa	$\uparrow$	16.949	< 0.001	$\uparrow$	29.947	< 0.001	$\uparrow$	54.803	< 0.001
Voat Askvoat	$\uparrow$	38.608	0.001	$\uparrow$	56.176	< 0.001	$\uparrow$	83.373	< 0.001
Voat Whatever	$\uparrow$	68.315	< 0.001	$\uparrow$	89.382	< 0.001	$\uparrow$	108.309	< 0.001
Youtube Carbonara	$\uparrow$	11.882	< 0.001	$\uparrow$	24.988	< 0.001	$\uparrow$	22.925	< 0.001
Youtube Football	$\uparrow$	11.946	< 0.001	$\uparrow$	39.510	< 0.001	$\uparrow$	76.700	< 0.001

Table S14: Agreement table between the classification given by Perspective API, and those of Detoxify and IMSYPP. Classification labels abbreviations: NT: non-toxic; T: toxic. Numbers represent percentages.

		Detoxify		IMSYPP	
Dataset	Perspective	NT	T	NT	T
Facebook Film	NT	94.00	6.00	89.27	10.73
	T	20.99	79.01	9.74	90.26
Facebook Sports	NT	92.53	7.47	85.13	14.87
	T	19.33	80.67	6.31	93.69
Reddit Askreddit	NT	94.58	5.42	88.75	11.25
	T	4.68	95.32	14.35	85.65
Reddit Iama	NT	96.61	3.39	90.18	9.82
	T	3.11	96.89	10.43	89.57
Reddit Movies	NT	94.76	5.24	85.74	14.26
	T	4.44	95.56	7.51	92.49
Telegram Crypto	NT	95.95	4.05	83.62	16.38
	T	8.35	91.65	9.38	90.62
Twitter Got	NT	97.61	2.39	96.28	3.72
	T	25.76	74.24	40.41	59.59
Twitter Nasa	NT	98.42	1.58	94.75	5.25
	T	21.71	78.29	30.71	69.29
Voat Askvoat	NT	92.26	7.74	80.52	19.48
	T	2.48	97.52	8.71	91.29
Voat Whatever	NT	90.33	9.67	72.56	27.44
	T	2.63	97.37	7.17	92.83
Youtube Carbonara	NT	97.26	2.74	97.54	2.46
	T	46.12	53.88	57.98	42.02
Youtube Football	NT	94.05	5.95	89.09	10.91
	T	17.29	82.71	8.11	91.89

Table S15: **Summary characteristics of Box-Plot shown in Fig. S3.** The table contains the sample size n (the number of conversations composed of $6-20$ comments), the lower whisker value l, the 25% percentile Q_1, the median Q_2, the 75% percentile Q_3, the upper whisker value u, the minimum m and maximum M value of each distribution.

Dataset	Type of comment	n	l	Q_1	Q_2	Q_3	u	m	M
Facebook Brexit	Begin	3234	0.000	0.032	0.100	0.239	0.544	0.000	0.939
Facebook Brexit	End	3234	0.000	0.038	0.113	0.271	0.618	0.000	0.962
Facebook News	Begin	74100	0.000	0.021	0.053	0.174	0.403	0.000	1.000
Facebook News	End	74100	0.000	0.022	0.062	0.199	0.463	0.000	0.988
Facebook Vaccines	Begin	128046	0.000	0.022	0.058	0.165	0.380	0.000	0.986
Facebook Vaccines	End	128046	0.000	0.022	0.065	0.173	0.399	0.000	0.988
Gab Feed	Begin	1342839	0.000	0.030	0.117	0.397	0.947	0.000	1.000
Gab Feed	End	1342839	0.000	0.027	0.111	0.378	0.903	0.000	1.000
Reddit Climate Change	Begin	1887	0.000	0.017	0.038	0.155	0.361	0.000	0.975
Reddit Climate Change	End	1887	0.000	0.017	0.039	0.119	0.270	0.000	0.950
Reddit Conspiracy	Begin	39711	0.000	0.025	0.052	0.125	0.275	0.000	0.988
Reddit Conspiracy	End	39711	0.000	0.023	0.054	0.203	0.473	0.000	0.982
Reddit News	Begin	5358	0.000	0.026	0.084	0.285	0.674	0.000	0.975
Reddit News	End	5358	0.000	0.025	0.083	0.285	0.674	0.000	0.961
Reddit Science	Begin	13758	0.000	0.020	0.027	0.075	0.159	0.000	0.950
Reddit Science	End	13758	0.000	0.018	0.033	0.094	0.206	0.000	0.911
Reddit Vaccines	Begin	4860	0.000	0.022	0.046	0.154	0.352	0.000	0.964
Reddit Vaccines	End	4860	0.000	0.022	0.050	0.158	0.361	0.000	0.975
Telegram Conspiracy	Begin	22239	0.000	0.018	0.053	0.243	0.578	0.000	0.982
Telegram Conspiracy	End	22239	0.000	0.018	0.054	0.285	0.686	0.000	0.982
Telegram News	Begin	23028	0.000	0.009	0.021	0.056	0.125	0.000	0.950
Telegram News	End	23028	0.000	0.007	0.019	0.067	0.155	0.000	0.964
Telegram Politics	Begin	33366	0.000	0.011	0.031	0.137	0.327	0.000	0.975
Telegram Politics	End	33366	0.000	0.009	0.029	0.137	0.330	0.000	0.975
Twitter Climate Change	Begin	59676	0.000	0.010	0.026	0.103	0.241	0.000	0.996
Twitter Climate Change	End	59676	0.000	0.010	0.027	0.110	0.260	0.000	0.986
Twitter News	Begin	52992	0.000	0.015	0.044	0.189	0.451	0.000	0.961
Twitter News	End	52992	0.000	0.016	0.051	0.224	0.536	0.000	0.965
Twitter Vaccines	Begin	74898	0.000	0.013	0.035	0.115	0.268	0.000	0.988
Twitter Vaccines	End	74898	0.000	0.013	0.034	0.114	0.267	0.000	1.000
Usenet Conspiracy	Begin	21708	0.000	0.049	0.119	0.289	0.644	0.000	0.982
Usenet Conspiracy	End	21708	0.000	0.046	0.119	0.305	0.687	0.000	0.982
Usenet News	Begin	45369	0.000	0.089	0.203	0.397	0.859	0.000	0.988
Usenet News	End	45369	0.000	0.075	0.202	0.398	0.878	0.000	0.988
Usenet Politics	Begin	173109	0.000	0.094	0.214	0.398	0.854	0.000	0.988
Usenet Politics	End	173109	0.000	0.081	0.204	0.399	0.870	0.000	0.988
Usenet Talk	Begin	121653	0.000	0.056	0.146	0.315	0.699	0.000	0.988
Usenet Talk	End	121653	0.000	0.053	0.141	0.323	0.726	0.000	0.989
Voat Conspiracy	Begin	112224	0.000	0.026	0.090	0.290	0.686	0.000	0.994
Voat Conspiracy	End	112224	0.000	0.024	0.083	0.305	0.725	0.000	0.988
Voat News	Begin	130953	0.000	0.039	0.180	0.470	0.989	0.000	0.989
Voat News	End	130953	0.000	0.037	0.184	0.479	0.989	0.000	0.989
Voat Politics	Begin	103344	0.000	0.039	0.177	0.462	0.994	0.000	0.994
Voat Politics	End	103344	0.000	0.039	0.192	0.479	0.988	0.000	0.988
Youtube Climate Change	Begin	5442	0.000	0.022	0.087	0.271	0.643	0.000	0.986
Youtube Climate Change	End	5442	0.000	0.024	0.103	0.277	0.656	0.000	0.985
Youtube News	Begin	66531	0.000	0.027	0.089	0.259	0.603	0.000	0.991
Youtube News	End	66531	0.000	0.029	0.098	0.275	0.643	0.000	0.991
Youtube Vaccines	Begin	9867	0.000	0.015	0.043	0.149	0.350	0.000	0.986
Youtube Vaccines	End	9867	0.000	0.016	0.050	0.164	0.386	0.000	0.986

Table S16: Summary characteristics of Box-Plot shown in Fig. S2. The table contains the sample size n, the lower whisker value l, the 25% percentile Q_1, the median Q_2, the 75% percentile Q_3, the upper whisker value u, the minimum m and maximum M value of each distribution.

Classificator	Type	n	l	Q_1	Q_2	Q_3	u	m	M
Detoxify	Toxicity	12	-0.002	0.003	0.009	0.010	0.011	-0.002	0.037
Detoxify	Participation	12	-0.107	-0.056	-0.030	-0.017	-0.009	-0.134	-0.009
Imsypp	Toxicity	12	-0.001	0.005	0.010	0.016	0.023	-0.038	0.072
Imsypp	Participation	12	-0.107	-0.056	-0.030	-0.017	-0.009	-0.134	-0.009
Perspective	Toxicity	12	0.000	0.004	0.006	0.008	0.008	-0.004	0.021
Perspective	Participation	12	-0.107	-0.056	-0.030	-0.017	-0.009	-0.134	-0.009

3 Supplementary Figures

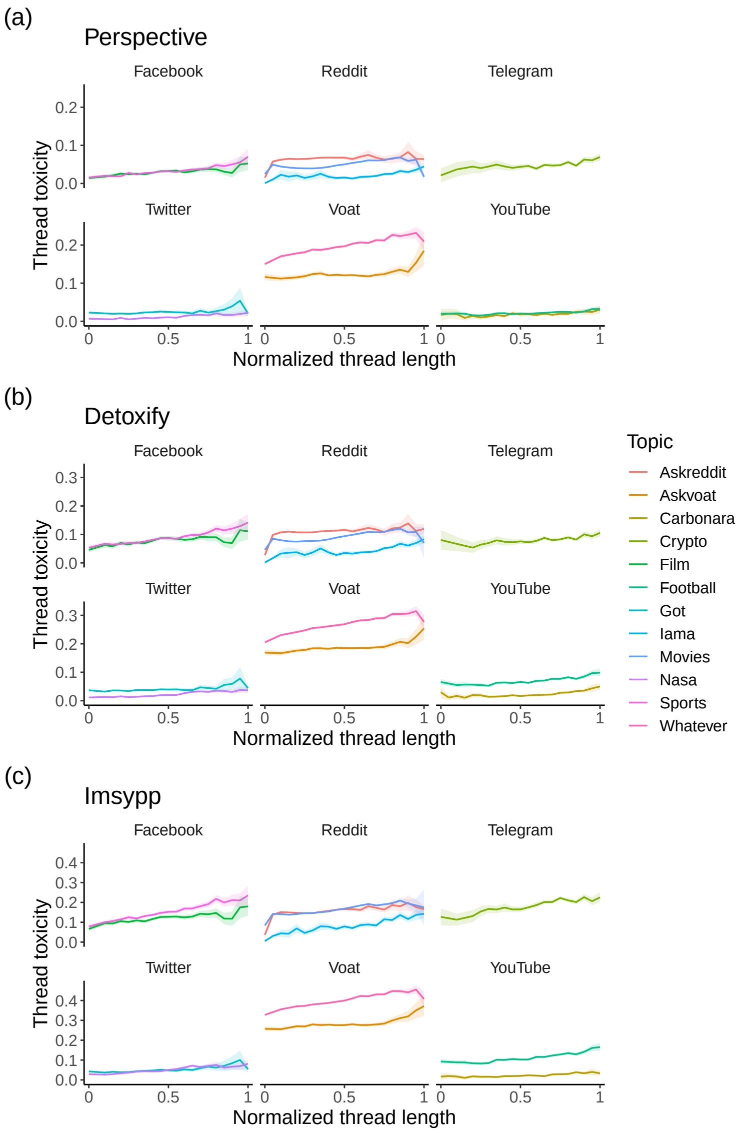

Fig. S1: **Validation dataset: toxicity increases with conversation size.** Mean fraction of toxic comments in conversations versus conversation size (see. Fig. 1), using Perspective API **a)**, Detoxify **b)** and IMSYPP **c)**. The sample sizes and the results of the relative Mann-Kendall tests are shown in Tab. S13. Trends are reported with their 95% confidence interval.

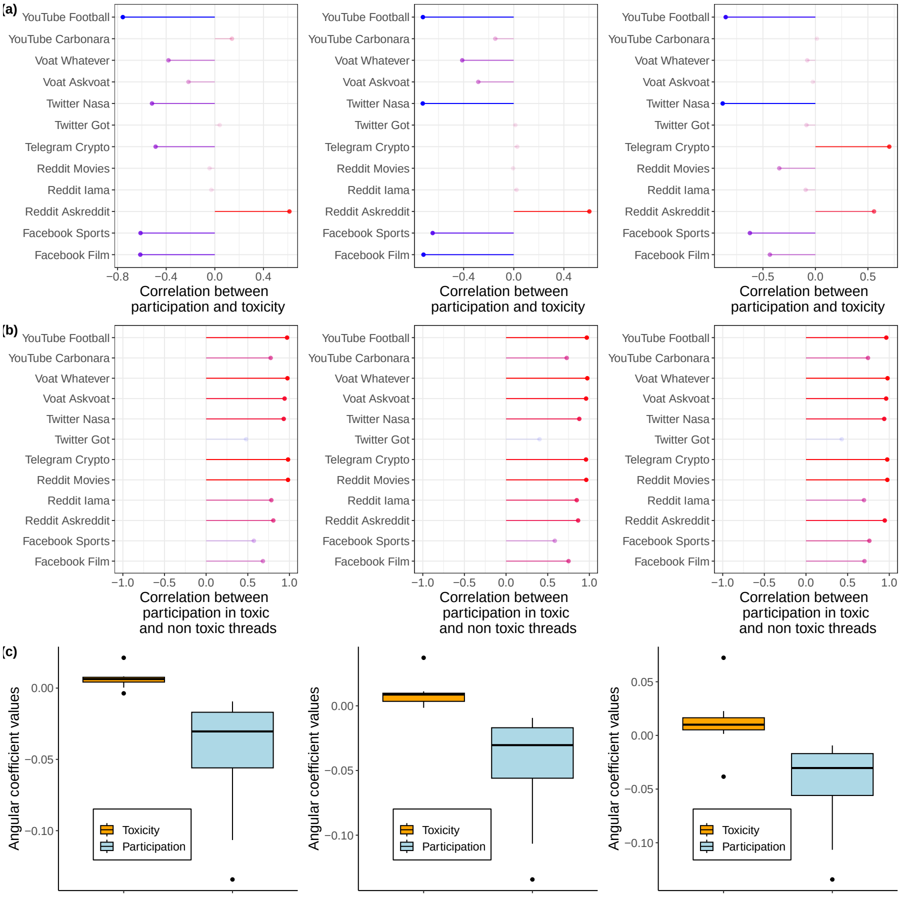

Fig. S2: **Participation analysis for the validation dataset.** (From left to right, results obtained with: Perspective API, Detoxify, IMSYPP) **a.** Pearson's correlation coefficients between user participation and toxicity trends for each dataset. **b.** Pearson's correlation coefficients between users' participation in toxic and non-toxic thread sets, for each dataset. **c.** Boxplots of the distribution of toxicity and participation trend slopes (N=12), as resulting from linear regression. The summary characteristics of each box plot are shown in Tab. S16

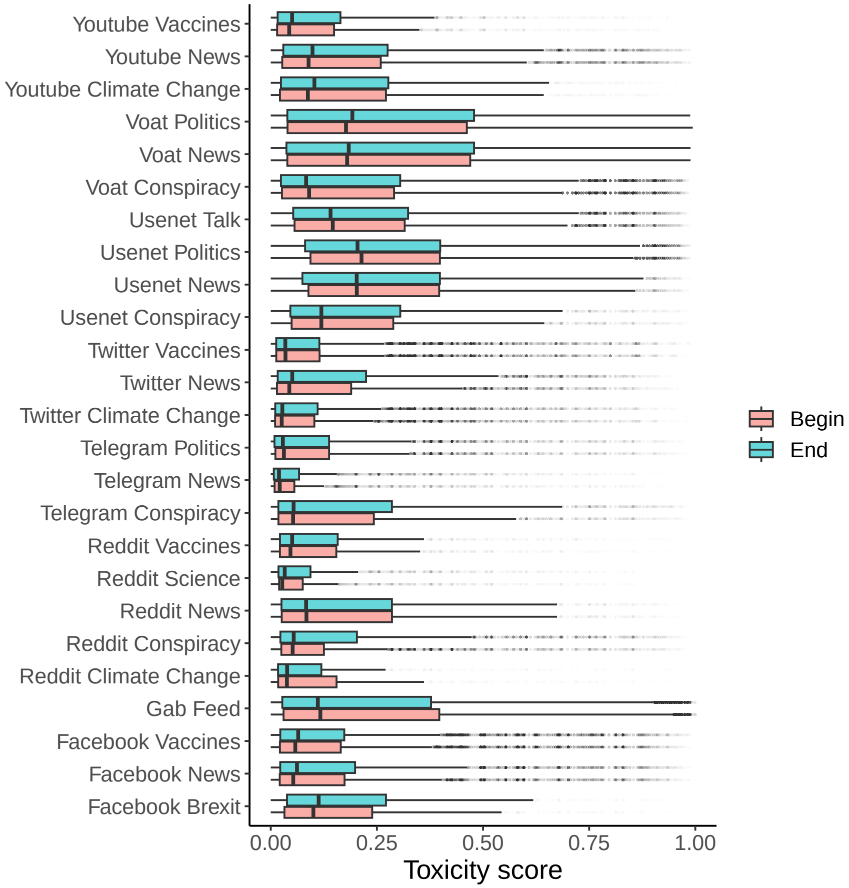

Fig. S3: **Short conversations are not short because of toxicity.** Each plot shows the box plot of the toxicity distribution for the first three ("begin" label) and last three comments ("end" label) of conversations composed of $6 - 20$ comments. No significant differences appear between the distributions. In general, the last comments are not more toxic than those preceding them. For Facebook news, a subsample containing 6.654.500 total comments was used. The summary characteristics of each box plot are shown in Tab. S15.

4 Software and Coding specifications

Here we report the specifications about the softwares employed and the coding process to develop this paper.

4.1 Softwares Employed

Code development was made with R 4.3.2, RStudio 2023.12.0-369, Python 3.10.11, Visual Studio Code 1.86.1, Docker Desktop 4.27.2 (137060)

4.2 List of R packages

Table S17: List of R packages with their own versions.

Package	Version
DBI	1.2.1
MASS	7.3-60
Matrix	1.6-5
MatrixModels	0.5-3
R6	2.5.1
RColorBrewer	1.1-3
RPostgreSQL	0.7-6
Rcpp	1.0.11
RcppEigen	0.3.3.9.4
RcppTOML	0.2.2
SparseM	1.81
abind	1.4-5
arrow	13.0.0.1
askpass	1.2.0
assertthat	0.2.1
backports	1.4.1
bit	4.0.5
bit64	4.0.5
boot	1.3-28.1
brio	1.1.4
broom	1.0.5
bursts	1.0-2
callr	3.7.3
car	3.1-2
carData	3.0-5
cli	3.6.1
clipr	0.8.0
colorspace	2.1-0
common	1.0.9
corrplot	0.92
cowplot	1.1.2
cpp11	0.4.6
crayon	1.5.2
curl	5.2.0

Table S17

Package	Version
data.table	1.14.8
datetime	0.1.4
desc	1.4.3
diffobj	0.3.5
digest	0.6.33
dplyr	1.1.3
ellipsis	0.3.2
evaluate	0.21
extraDistr	1.10.0
fansi	1.0.4
farver	2.1.1
fs	1.6.3
generics	0.1.3
ggplot2	3.4.3
ggpubr	0.6.0
ggrepel	0.9.5
ggsci	3.0.0
ggsignif	0.6.4
glue	1.6.2
gridExtra	2.3
gtable	0.3.4
here	1.0.1
hms	1.1.3
httr	1.4.7
isoband	0.2.7
jsonlite	1.8.7
labeling	0.4.3
lattice	0.21-8
lifecycle	1.0.3
lme4	1.1-35.1
log4r	0.4.3
logr	1.3.4
lubridate	1.9.2
magrittr	2.0.3
mgcv	1.8-42
mime	0.12
minqa	1.2.6
munsell	0.5.0
ndjson	0.9.0
nlme	3.1-162
nloptr	2.0.3
nnet	7.3-19

Table S17

**Package**	**Version**
numDeriv	2016.8-1.1
openssl	2.1.1
patchwork	1.2.0
pbkrtest	0.5.2
pillar	1.9.0
pkgbuild	1.4.3
pkgconfig	2.0.3
pkgload	1.3.4
plyr	1.8.8
png	0.1-8
polynom	1.4-1
praise	1.0.0
prettyunits	1.2.0
processx	3.8.3
progress	1.2.2
ps	1.7.6
purrr	1.0.2
quantreg	5.97
rappdirs	0.3.3
readr	2.1.4
rematch2	2.1.2
renv	1.0.3
reticulate	1.34.0
rlang	1.1.1
rprojroot	2.0.4
rstatix	0.7.2
scales	1.2.1
stringi	1.7.12
stringr	1.5.0
survival	3.5-7
sys	3.4.2
testthat	3.2.1
tibble	3.2.1
tidyr	1.3.0
tidyselect	1.2.0
timechange	0.2.0
trend	1.1.6
triebear	0.4.1
tzdb	0.4.0
urltools	1.7.3
utf8	1.2.3
vctrs	0.6.3

Table S17

Package	Version
viridisLite	0.4.2
vroom	1.6.3
waldo	0.5.2
withr	2.5.0
xtable	1.8-4

References

[1] Schmidt, A., Zollo, F., Scala, A. & Quattrociocchi, W. Polarization rank: A study on european news consumption on facebook (2018). Preprint at https://arxiv.org/abs/1805.08030.
