# Supplementary material for: Persistent interaction patterns across social media platforms and over time
Source: Nature. 2024 Mar 20;628(8008):582–9. doi: 10.1038/s41586-024-07229-y (PMC11023927; doi:10.1038/s41586-024-07229-y)
Supplement: Supplementary file 2 — Reporting Summary [file 41586_2024_7229_MOESM2_ESM.pdf]

nature portfolio

Corresponding author(s): Walter Quattrociocchi, Matteo Cinelli

Last updated by author(s): Feb 16th, 2024

Reporting Summary

Nature Portfolio wishes to improve the reproducibility of the work that we publish. This form provides structure for consistency and transparency in reporting. For further information on Nature Portfolio policies, see our Editorial Policies and the Editorial Policy Checklist.

Statistics

For all statistical analyses, confirm that the following items are present in the figure legend, table legend, main text, or Methods section.

n/a	Confirmed	
☐	☒	The exact sample size (*n*) for each experimental group/condition, given as a discrete number and unit of measurement
☐	☒	A statement on whether measurements were taken from distinct samples or whether the same sample was measured repeatedly
☐	☒	The statistical test(s) used AND whether they are one- or two-sided<br>*Only common tests should be described solely by name; describe more complex techniques in the Methods section.*
☐	☒	A description of all covariates tested
☐	☒	A description of any assumptions or corrections, such as tests of normality and adjustment for multiple comparisons
☐	☒	A full description of the statistical parameters including central tendency (e.g. means) or other basic estimates (e.g. regression coefficient) AND variation (e.g. standard deviation) or associated estimates of uncertainty (e.g. confidence intervals)
☐	☒	For null hypothesis testing, the test statistic (e.g. *F*, *t*, *r*) with confidence intervals, effect sizes, degrees of freedom and *P* value noted<br>*Give P values as exact values whenever suitable.*
☒	☐	For Bayesian analysis, information on the choice of priors and Markov chain Monte Carlo settings
☒	☐	For hierarchical and complex designs, identification of the appropriate level for tests and full reporting of outcomes
☐	☒	Estimates of effect sizes (e.g. Cohen's *d*, Pearson's *r*), indicating how they were calculated

Our web collection on statistics for biologists contains articles on many of the points above.

Software and code

Policy information about availability of computer code

Data collection	Datasets from Facebook, Twitter, Gab and Voat were obtained from the previous literature. Reddit dataset was obtained through pushshift.io, therefore no software nor code were necessary. Telegram data was collected with the following tool available at:<br><br>The entire repository can be accessed at the following DOI: 10.17605/OSF.IO/FQ5DY
Data analysis	Data analysis were performed by running scripts with R 4.3.2, and Python 3.10.11, which are available in the paper repository.

For manuscripts utilizing custom algorithms or software that are central to the research but not yet described in published literature, software must be made available to editors and reviewers. We strongly encourage code deposition in a community repository (e.g. GitHub). See the Nature Portfolio guidelines for submitting code & software for further information.

Data

Policy information about availability of data

All manuscripts must include a data availability statement. This statement should provide the following information, where applicable:

- Accession codes, unique identifiers, or web links for publicly available datasets
- A description of any restrictions on data availability
- For clinical datasets or third party data, please ensure that the statement adheres to our policy

Facebook: we employed datasets from previous works collected using Facebook Graph API.

List of works:
Schmidt, A. L., Zollo, F., Scala, A., Betsch, C. & Quattrociocchi, W. Polarization of the vaccination debate on facebook. Vaccine 36, 3606– 960 3612 (2018)
Schmidt, A. L. et al. Anatomy of news consumption on facebook. Proceedings of the National Academy of Sciences 114, 3035–3039 (2017).
Del Vicario, M., Zollo, F., Caldarelli, G., Scala, A. & Quattrociocchi, W. 964 Mapping social dynamics on facebook: The brexit debate. Social Networks 965 50, 6–16 (2017).
Schmidt, A., Zollo, F., Scala, A. & Quattrociocchi, W. Polarization rank: A study on european news consumption on facebook (2018). Preprint at https://arxiv.org/abs/1805.08030

Gab: we collected data from Pushshift.io archive;

Reddt: we collected data from Pushshift.io archive;

Telegram: data were manually downloaded using Telegram Web service;

Twitter: we collected data using Twitter Academic API;
List of works:
Valensise, C. M. et al., Lack of evidence for correlation between covid-19 infodemic and vaccine acceptance infodemic and vaccine acceptance;
Falkenberg, M. et al. Growing polarization around climate change on social media. Nature Climate Change 1–8 (2022)
Quattrociocchi, A., Etta, G., Avalle, M., Cinelli, M. & Quattrociocchi, W. Reliability of news and toxicity in Twitter conversations. In Hopfgartner, F., Jaidka, K., Mayr, P., Jose, J. & Breitsohl, J. (eds.) Social Informatics, 245–256 (Springer International Publishing, Cham, 2022)

Usenet: we collected data by querying the Usenet Archive;

Voat: we employed a dataset presented in another paper;
List of work:
Mekacher, A. & Papasavva, A. “I can’t keep it up” a dataset from the defunct voat.co news aggregator. Proceedings of the International AAAI,Conference on Web and Social Media,16, 1302–1311 (2022)
YouTube: we employed a dataset from previous works collected using YouTube Data API and new data collected using YouTube Data API.
List of work:
Falkenberg, M. et al. Growing polarization around climate change on social media. Nature Climate Change 1–8 (2022)

We collected data from R ver. 3.* and Python 3.*.

Research involving human participants, their data, or biological material

Policy information about studies with human participants or human data. See also policy information about sex, gender (identity/presentation), and sexual orientation and race, ethnicity and racism.

Reporting on sex and gender	No information about sex and gender was collected.
Reporting on race, ethnicity, or other socially relevant groupings	No information about race, ethnicity or other socially relevant groupings was collected.
Population characteristics	No demographic information about the collected users were retained.
Recruitment	Our selection of platforms, time periods, and specific posts or individuals was driven by the goal of assembling the most representative dataset possible. This was to ensure a comprehensive understanding of online conversation dynamics. We focused on achieving broad heterogeneity in our data, capturing diverse voices and perspectives across various social media ecosystems. This approach was critical for analyzing the nuances of digital discourse, enabling us to explore a wide array of interactions and trends within online communities.
Ethics oversight	Our study involved the analysis of publicly available social media data, focusing on aggregate trends and anonymized interactions without targeting or identifying individual users. Given the public nature of the data and our adherence to ethical guidelines that protect individual privacy, our research did not require Institutional Review Board (IRB) oversight. We ensured compliance with all relevant social media platforms' terms of service and data use policies, and our methodology was designed to respect user privacy and data protection principles inherently.

Note that full information on the approval of the study protocol must also be provided in the manuscript.

Field-specific reporting

Please select the one below that is the best fit for your research. If you are not sure, read the appropriate sections before making your selection.

☐ Life sciences ☒ Behavioural & social sciences ☐ Ecological, evolutionary & environmental sciences

For a reference copy of the document with all sections, see nature.com/documents/nr-reporting-summary-flat.pdf

Behavioural & social sciences study design

All studies must disclose on these points even when the disclosure is negative.

Study description	We perform a quantitative study based on social media data analysis using a statistical approach. We also employed Deep Learning Tools to label our data.
Research sample	The research sample collected for the study includes several topics considered divisive and, therefore, with the potential to exhibit toxicity dynamics in a clearer way.<br><br>List of works that used our data:<br>Schmidt, A. L., Zollo, F., Scala, A., Betsch, C. & Quattrociocchi, W. Polarization of the vaccination debate on facebook. Vaccine 36, 3606– 960 3612 (2018)<br>Schmidt, A. L. et al. Anatomy of news consumption on facebook. Proceedings of the National Academy of Sciences 114, 3035–3039 (2017).<br>Del Vicario, M., Zollo, F., Caldarelli, G., Scala, A. & Quattrociocchi, W. 964 Mapping social dynamics on facebook: The brexit debate. Social Networks 965 50, 6–16 (2017).<br>Valensise, C. M. et al., Lack of evidence for correlation between covid-19 infodemic and vaccine acceptance infodemic and vaccine acceptance;<br>Falkenberg, M. et al. Growing polarization around climate change on social media. Nature Climate Change 1–8 (2022)<br>Quattrociocchi, A., Etta, G., Avalle, M., Cinelli, M. & Quattrociocchi, W. Reliability of news and toxicity in Twitter conversations. In Hopfgartner, F., Jaidka, K., Mayr, P., Jose, J. & Breitsohl, J. (eds.) Social Informatics, 245–256 (Springer International Publishing, Cham, 2022)<br>Mekacher, A. & Papasavva, A. "I can't keep it up" a dataset from the defunct voat.co news aggregator. Proceedings of the International AAAI,Conference on Web and Social Media,16, 1302–1311 (2022)
Sampling strategy	We used a keyword search for collecting data adherent to certain topics. Therefore, we employed all the data obtained from such a process.<br>For two news datasets we collected data from profiles listed by the Europe Media Monitor and Newsguard.
Data collection	Facebook: we employed datasets from previous works collected using Facebook Graph API.<br>List of works:<br>Schmidt, A. L., Zollo, F., Scala, A., Betsch, C. & Quattrociocchi, W. Polarization of the vaccination debate on facebook. Vaccine 36, 3606– 960 3612 (2018)<br>Schmidt, A. L. et al. Anatomy of news consumption on facebook. Proceedings of the National Academy of Sciences 114, 3035–3039 (2017).<br>Del Vicario, M., Zollo, F., Caldarelli, G., Scala, A. & Quattrociocchi, W. 964 Mapping social dynamics on facebook: The brexit debate. Social Networks 965 50, 6–16 (2017).<br>Schmidt, A., Zollo, F., Scala, A. & Quattrociocchi, W. Polarization rank: A study on european news consumption on facebook (2018). Preprint at https://arxiv.org/abs/1805.08030<br><br>Gab: we collected data from Pushshift.io archive;<br>Reddt: we collected data from Pushshift.io archive;<br>Telegram: data were manually downloaded using Telegram Web service;<br>Twitter: we collected data using Twitter Academic API;<br>List of works:<br>Valensise, C. M. et al., Lack of evidence for correlation between covid-19 infodemic and vaccine acceptance infodemic and vaccine acceptance;<br>Falkenberg, M. et al. Growing polarization around climate change on social media. Nature Climate Change 1–8 (2022)<br>Quattrociocchi, A., Etta, G., Avalle, M., Cinelli, M. & Quattrociocchi, W. Reliability of news and toxicity in Twitter conversations. In Hopfgartner, F., Jaidka, K., Mayr, P., Jose, J. & Breitsohl, J. (eds.) Social Informatics, 245–256 (Springer International Publishing, Cham, 2022)<br><br>Usenet: we collected data by querying the Usenet Archive;<br>Voat: we employed a dataset presented in another paper;<br>List of work:<br>Mekacher, A. & Papasavva, A. "I can't keep it up" a dataset from the defunct voat.co news aggregator. Proceedings of the International AAAI,Conference on Web and Social Media,16, 1302–1311 (2022)<br>YouTube: we employed a dataset from previous works collected using YouTube Data API and new data collected using YouTube Data API.<br>List of work:<br>Falkenberg, M. et al. Growing polarization around climate change on social media. Nature Climate Change 1–8 (2022)<br><br>We collected data from R ver. 3.* and Python 3.*.
Timing	Facebook Brexit & 2015-12-31 - 2016-07-29 & 464764 & 4241 & 252156 & 0.06 \\ Facebook News & 2009-09-09 - 2016-08-18 & 362718451 & 6898312 & 60235461 & 0.06 \\ Facebook Vaccines & 2010-01-02 - 2017-07-17 & 2064980 & 153137 & 387084 & 0.04 \\ Gab Feed & 2016-08-10 - 2018-10-29 & 14641433 & 3764443 & 166833 & 0.13 \\ Reddit Climate Change & 2018-01-01 - 2022-12-12 & 70648 & 5057 & 26521 & 0.07 \\ Reddit Conspiracy & 2018-01-01 - 2022-12-08 & 777393 & 35092 & 92678 & 0.07 \\ Reddit News & 2018-01-01 - 2018-12-31 & 389582 & 7798 & 109860 & 0.09 \\ Reddit Science & 2018-01-01 - 2022-12-11 & 549543 & 28330 & 211546 & 0.01 \\ Reddit Vaccines & 2018-01-01 - 2022-11-06 & 66457 & 4539 & 5192 & 0.04 \\ Telegram Conspiracy & 2019-08-30 - 2022-12-20 & 1416482 & 32592 & 150251 & 0.12 \\ Telegram News & 2018-04-09 -

2022-12-20 & 724482 & 28288 & 16716 & 0.02 \\ Telegram Politics & 2017-08-04 - 2022-12-19 & 491294 & 27749 & 6132 & 0.04 \\ Twitter Climate Change & 2020-01-01 - 2023-01-10 & 9709855 & 130136 & 3577890 & 0.07 \\ Twitter News & 2020-01-01 - 2022-11-29 & 9487587 & 97797 & 1710213 & 0.07 \\ Twitter Vaccines & 2010-01-23 - 2023-01-25 & 49437212 & 125667 & 11857050 & 0.08 \\ Usenet Conspiracy & 1994-09-01 - 2005-12-30 & 284838 & 72655 & 48224 & 0.05 \\ Usenet News & 1992-12-05 - 2005-12-31 & 621084 & 169036 & 76620 & 0.09 \\ Usenet Politics & 1992-06-29 - 2005-12-31 & 2657772 & 625945 & 209905 & 0.08 \\ Usenet Talk & 1989-02-13 - 2005-12-31 & 2103939 & 328009 & 156542 & 0.06 \\ Voat Conspiracy & 2018-01-09 - 2020-12-25 & 1024812 & 99953 & 27641 & 0.10 \\ Voat News & 2013-11-21 - 2020-12-25 & 1397955 & 170801 & 88434 & 0.19 \\ Voat Politics & 2014-06-16 - 2020-12-25 & 1083932 & 143103 & 66424 & 0.19 \\ Youtube Climate Change & 2014-03-16 - 2022-02-28 & 846300 & 9022 & 436246 & 0.06 \\ Youtube News & 2006-02-13 - 2022-02-08 & 20536162 & 107880 & 4310827 & 0.07 \\ Youtube Vaccines & 2020-01-31 - 2021-10-24 & 2648909 & 14147 & 902340 & 0.04 \\

Data exclusions: No data were excluded.

Non-participation: Due to the usage of extant data, no participants could drop out.

Randomization: We performed permutation test of data.

Reporting for specific materials, systems and methods

We require information from authors about some types of materials, experimental systems and methods used in many studies. Here, indicate whether each material, system or method listed is relevant to your study. If you are not sure if a list item applies to your research, read the appropriate section before selecting a response.

Materials & experimental systems

n/a	Involved in the study
☒	☐ Antibodies
☒	☐ Eukaryotic cell lines
☒	☐ Palaeontology and archaeology
☒	☐ Animals and other organisms
☒	☐ Clinical data
☒	☐ Dual use research of concern
☒	☐ Plants

Methods

n/a	Involved in the study
☒	☐ ChIP-seq
☒	☐ Flow cytometry
☒	☐ MRI-based neuroimaging

Plants

Seed stocks: No plants materials were used.

Novel plant genotypes: No novel plant genotypes were used.

Authentication: Seed stocks or novel plant genotypes were used.
